# The RXR Agonist MSU-42011 Reduces Tumor Burden in a Murine Preclinical NF1-Deficient Model

**DOI:** 10.3390/cancers17121920

**Published:** 2025-06-09

**Authors:** Pei-Yu Hung, Jessica A. Moerland, Ana S. Leal, Bilal Aleiwi, Edmund Ellsworth, D. Wade Clapp, Verena Staedtke, Renyuan Bai, Karen T. Liby

**Affiliations:** 1 Department of Physiology, Michigan State University, East Lansing, MI 48824, USA; hungpeiy@msu.edu; 2 Department of Pediatrics and Herman B Wells Center for Pediatric Research, Indiana University School of Medicine, Indianapolis, IN 46202, USA; jamoerla@iu.edu (J.A.M.); dclapp@iu.edu (D.W.C.); 3 Department of Pharmacology and Toxicology, Michigan State University, East Lansing, MI 48824, USA; aleiwibi@msu.edu (B.A.); ellswo59@msu.edu (E.E.); ktliby@iu.edu (K.T.L.); 4 Department of Medicine, Divisions of Hematology/Oncology, Indiana University School of Medicine, Indianapolis, IN 46202, USA; 5 Department of Microbiology and Immunology, Indiana University School of Medicine, Indianapolis, IN 46202, USA; 6 Department of Biochemistry and Molecular Biology, Indiana University School of Medicine, Indianapolis, IN 46202, USA; 7 Department of Medical and Molecular Genetics, Indiana University School of Medicine, Indianapolis, IN 46202, USA; 8 Department of Neurology, Johns Hopkins University, Baltimore, MD 21287, USA; vstaedt1@jhmi.edu; 9 Kennedy Krieger Institute, Baltimore, MD 21205, USA; rbai1@jhmi.edu; 10 Department of Neurosurgery, Johns Hopkins University, Baltimore, MD 21287, USA

**Keywords:** neurofibromatosis type 1, plexiform neurofibromas (PNFs), malignant peripheral nerve sheath tumors (MPNSTs), retinoid X receptor (RXR) agonist, selumetinib

## Abstract

**Background/Objectives:** Neurofibromatosis type 1 (NF1) is a prevalent inherited disorder, with approximately 50% of affected individuals developing plexiform neurofibromas (PNFs), which can progress to highly aggressive malignant peripheral nerve sheath tumors (MPNSTs). While selumetinib is FDA-approved for PNFs, its efficacy in MPNSTs is limited and associated with dose-limiting toxicities. *NF1* deficiency drives tumorigenesis and alters immune dynamics via RAS hyperactivation. Given the substantial macrophage infiltration in NF1 lesions and its association with disease progression, we hypothesized that targeting tumor-promoting immune cells with the retinoid X receptor (RXR) agonist MSU-42011 could be an alternative therapeutic strategy, as it has shown promise in KRAS-driven cancers by decreasing pERK levels and reducing tumor-promoting immune cells. **Methods:** We examined the effects of MSU-42011 and selumetinib, alone and in combination, on NF1-deficient cells and in a syngeneic MPNST model. **Results:** In vivo, the combination of MSU-42011 and selumetinib significantly reduced tumor growth, pERK levels, and tumor-promoting macrophages and increased activated CD8^+^ T cells in syngeneic MPNST models. In NF1-deficient cells, MSU-42011 or selumetinib reduced pERK levels, with combination treatment achieving greater reductions. Conditioned media (CM) from NF1-deficient cells increased the protein and mRNA levels of several cytokines and chemokines in human THP1 cells and bone marrow-derived macrophages (BMDMs). MSU-42011 and selumetinib, alone or in combination, partially reversed this induction. **Conclusions:** These findings suggest RXR agonists may have therapeutic potential against NF1, and their combination with MEK inhibitors could represent a promising strategy for NF1-associated tumors. Further studies are needed to validate these results and assess their translational relevance.

## Simple Summary

Neurofibromatosis type 1 (NF1) is a genetic disorder that leads to the formation of plexiform neurofibromas (PNFs), which can progress to malignant peripheral nerve sheath tumors (MPNSTs), a major cause of death in individuals with NF1. Current treatments, like the MEK inhibitor selumetinib, are limited to PNFs and have significant side effects, highlighting the urgent need for more effective therapies. Our research identified MSU-42011, a retinoid X receptor (RXR) agonist, as a promising candidate in preclinical NF1-deficient models. MSU-42011 reduced tumor growth, pERK levels, and tumor-promoting immune cells, while increasing activated T cells; further tumor reduction was observed when combined with selumetinib. The combination therapy also suppressed pro-tumor cytokine signaling, suggesting potential for immune modulation. These findings highlight RXR agonists as an effective therapeutic strategy for NF1, with combination therapy offering a promising treatment option.

## 1. Introduction

Neurofibromatosis type 1 (NF1) is an autosomal dominant genetic disease with an incidence of 1 in 3000 individuals [1]. It is caused by germline mutations of the *NF1* tumor suppressor gene, which encodes neurofibromin, a GTPase-activating protein (GAP) for p21RAS [2,3,4]. Loss of *NF1* results in a hyperactive RAS signaling pathway and its downstream cascades, including the MAPK pathway (RAF/MEK/ERK), which promotes abnormal cell proliferation and neoplasia [2,3,4]. Plexiform neurofibromas (PNFs), a hallmark manifestation of *NF1* loss, are benign nerve and soft tissue tumors that develop in about 20–50% of patients [5]. These tumors can arise in various locations, including the eyes and skin. While these lesions are typically not medically serious, PNFs can significantly impact the patient’s quality of life due to disfigurement, pain, and functional impairment [6]. Although PNFs are among the most recognizable features of NF1, more clinically severe tumors in neurofibromatosis include trigeminal and vestibular schwannomas, as well as high-grade IDH-mutant astrocytomas in the posterior cranial fossa. These tumors present major therapeutic challenges due to their complex locations and risk of neurological deficits, as highlighted in a recent clinical series emphasizing long-term functional outcomes and recurrence risks following surgical and multimodal interventions [7,8]. Additionally, about 10% of PNFs can progress to highly aggressive malignant peripheral nerve sheath tumors (MPNSTs), a leading cause of death in patients with *NF1* loss [5]. Currently, no effective drugs are available to treat MPNSTs; moreover, these tumors respond poorly to chemotherapy and exhibit high rates of recurrence [9,10]. Consequently, the prognosis for patients with MPNST remains poor, with a 5-year survival rate of approximately 20% to 50% [11,12]. The malignant transformation of PNF often occurs through intermediate lesions known as atypical neurofibroma or atypical neurofibromatous neoplasms of uncertain biological potential (ANF/ANNUBP), which are characterized by distinct histopathologic features and *CDKN2A* copy number loss, serving as a critical transition step between PNFs and MPNSTs [13,14]. While surgical resection can partially remove tumors, complete resection is rarely possible due to the infiltrative nature of these tumors [9].

Currently, selumetinib is an FDA-approved MEK1/2 inhibitor for treating symptomatic, inoperable PNF in pediatric patients with NF1 [15]. Oral administration of selumetinib inhibits ERK phosphorylation and reduces the number, volume, and proliferation of PNFs [16,17]. However, some patients either do not respond to MEK inhibitor monotherapy, including selumetinib, binimetinib, and trametinib [18,19,20], or eventually develop MPNST within a few months of treatment, indicating that MEK inhibitor monotherapy may be insufficient to prevent malignant transformation [21]. Moreover, selumetinib has toxic effects, including acneiform rash, gastrointestinal disturbance, and asymptomatic creatine kinase elevation [16]. As such, there remains an unmet need for new NF1 treatment strategies that bypass resistance or reduce toxicity.

Deficiency of the *NF1* gene not only promotes tumorigenesis but also has broad effects on the immune cells and cytokine signaling driven by the hyperactive RAS signaling pathway [22,23,24]. Although immune cells are rare in healthy peripheral nerves, up to 30% of the cells in PNF and 50% of the cells in MPNST are macrophages [24], with macrophage recruitment correlating with disease progression [25,26]. These neurofibroma-associated macrophages play a key role in shaping the tumor microenvironment (TME) by recruiting T cells and other immune cells, contributing to an immunosuppressive milieu [24]. While the direct role of T cells in neurofibromatosis has yet to be fully explored, recent studies demonstrate that T cells are important for sustaining macrophage recruitment within the TME, promoting NF1 tumor initiation and growth [27]. Preclinical studies have shown that the combination of cyclin-dependent kinase 4/6 (CDK4/6) and MEK inhibitors can improve the responsiveness of MPNST to anti-PD-L1 immune checkpoint blockade (ICB) [28]. And several clinical trials of ICB in MPNSTS are currently underway, including a phase I study (NCT04465643) assessing the use of neoadjuvant nivolumab (PD-1 inhibitor) in combination with ipilimumab (CTLA-4 inhibitor) in patients with newly diagnosed ANNUBP and MPNST. These studies demonstrate the potential of immunotherapy in a subset of NF1-associated MPNSTs, and the combination treatment offers a promising approach to address some of these gaps, providing more effective and comprehensive solutions compared to existing therapies.

Several retinoid X receptor (RXR) agonists modulate immune cells in the TME and reduce tumor growth in *Kras*-mutated cancers [29,30]. As with *NF1* mutations [23,24], *KRAS* mutations increase tumor-promoting immune cell function in the TME [31,32], which suggests these compounds offer a promising new therapeutic strategy for PNF and MPNST [33]. RXR agonists are ligands that bind to and activate retinoid X receptors. Upon binding, RXR dimerizes with various receptors, acting as transcription factors for genes involved in several biological processes, including immune regulation [34]. In a carcinogenesis-induced Kras-driven lung cancer, the novel RXR agonist MSU-42011 decreased tumor burden by reducing tumor-promoting CD206^+^ macrophages, immunosuppressive CD4-FOXP3 (Tregs), which led to increasing activated cytotoxic T cells [29]. Furthermore, MSU-42011 decreased pERK levels within the lung tumors [29]. The immunomodulatory and anti-tumor effects of MSU-42011 were also found in a HER2^+^ breast cancer model [29]. Despite the efficacy of RXR agonists for the reduction of tumor-promoting immune cells in the TME and inhibition of tumor progression [29,30,33,35], no RXR agonist has been directly tested in clinical trials or preclinical studies for PNFs or MPNSTs. Due to the similarities between NF1- and Kras-driven cancers, including a hyperactive RAS signaling pathway and aberrant inflammatory signaling, we tested MSU-42011 in relevant preclinical models of NF1.

In this study, we assessed the effects of MSU-42011, selumetinib, and combinations using in vitro, ex vivo, and in vivo models of NF1. In murine and human NF1-deficient cells, combination treatment with MSU-42011 and selumetinib decreased pERK levels. It also partially inhibited the elevated cytokine and chemokine levels in macrophages treated with conditioned media (CM) from NF1-deficient cells. Moreover, in an immunocompetent mouse model of MPNST, the combination treatment of MSU-42011 and selumetinib reduced pERK levels, CD206^+^ cells, and tumor growth, further than single treatments. These data demonstrate the immunomodulatory and anti-tumor effects of MSU-42011 alone or in combination with selumetinib and support their potential use as a combination therapy for the treatment of PNFs and MPNSTs in patients with NF1.

## 2. Materials and Methods

### 2.1. Drugs

MSU-42011 was synthesized (>95% purity) as described [30]. Selumetinib (>99% purity) and bexarotene (>99% purity) were purchased from LC Laboratories (Woburn, MA, USA).

### 2.2. Cell Culture

#### 2.2.1. Culture of Human Immortalized NF1^−/−^ Schwann Cells, Murine Nf1-Related MPNSTs, and Murine Lewis Lung Carcinoma Cells (LL2)

Human immortalized normal Schwann cells (ipn02.3 2λ), non-tumor Schwann cells with *NF1* mutation (ipnNF95.11c) and PNF cells (ipNF95.11bC and ipNF95.6), and murine LL2 lung cancer cells, a Kras/Nras mutant lung cancer model [36], were purchased from ATCC (Manassas, VA, USA). The human immortalized NF1^−/−^ Schwann cells were used in the preclinical evaluation of targeted therapies for NF1-associated PNF, including selumetinib [37,38]. Primary murine Nf1-related MPNSTs from a *cis* Nf1;Tp53 C57BL/6 mice were provided by Verena Staedtke and Renyuan Bai lab (Baltimore, MD, USA) [39]. The addition of heterozygous Tp53 knockout accelerates cancer development, mimicking mutations required for malignant transformation, and has been used to study Nf1-related MPNSTs [39]. Cells were cultured at 37 °C and 5% CO_2_ in DMEM media (Corning Cellgro, Manassas, VA, USA) containing FBS (10%, Corning Cellgro) and penicillin/streptomycin (1%, Corning Cellgro). Trypsin-EDTA (0.25%, Corning Cellgro) was used to dissociate cells for passaging upon reaching confluence.

#### 2.2.2. Culture of Primary Murine Nf1 Cdkn2a^−/−^ and Nf1^−/−^ DNSCs

Primary murine dorsal root ganglia (DRG)/neurosphere cells (DNSCs) isolated from Nf1^flox/flox^ and Nf1^flox/flox^ Cdkn2a^flox/flox^ genotypes were obtained from Wade Clapp lab (Indianapolis, IN, USA) [13,40]. These cells contain the neural crest-derived tumorigenic cell of origin for PNFs [41] and have been used in multiple studies modeling PNF initiation, malignant progression, and the molecular mechanisms driving NF1-related tumorigenesis [13,40]. Cells were cultured at 37 °C and 5% CO_2_ in DNSC complete media. DNSC complete media was made with serum-free DMEM/F12 media (Gibco) containing HEPES (1 M, Gibco, Grand Island, NY, USA), heparin (0.2%, StemCell, Vancouver, BC, Canada), glucose (30%, Sigma, St. Louis, MO, USA), sodium bicarbonate (7.5%, Gibco), N2 supplement (1%, Gibco), glutamine (1%, Gibco), sodium pyruvate (1%, Gibco), B27 (without vitamin A, 2%, Gibco), epidermal growth factor (20 ng/mL, Sigma), basic fibroblast growth factor (40 ng/mL, PeproTech, Cranbury, NJ, USA), penicillin/streptomycin (1%, Corning Cellgro), and amphotericin B/fungizone (1 μg/mL, Gibco). Nf1^flox/flox^ DNSCs were transiently infected with a GFP-Cre adenovirus (1:500, University of Iowa) for 24 h to delete the Nf1 floxed alleles (served as PNF precursor; Nf1^f/f^ Cre+) or GFP adenovirus (1:500, University of Iowa, Iowa City, IA, USA) as a control (DNSC without *Nf1* mutation; Nf1^f/f^ Cre-). Fluorescence microscopy was used to observe the efficiency of virus transduction (Figure S1A). Efficient Cre-mediated excision of floxed Nf1 alleles and the corresponding loss of neurofibromin expression were confirmed by Western blot (Figure S1B). Unlike Nf1^flox/flox^ DNSCs, Nf1^flox/flox^ Cdkn2a^flox/flox^ DNSCs were already infected with Cre recombinase (served as MPNST precursors) and confirmed by polymerase chain reaction (PCR) in the Clapp lab before we obtained the cells [13]. TrypLE express enzyme (1×, Gibco) was used to dissociate cells for passaging upon reaching confluence.

#### 2.2.3. Culture and Differentiation of Human THP1 Cells and BMDMs

A human THP1 monocyte was purchased from ATCC. Bone marrow-derived macrophages (BMDMs) were isolated from C57BL/6 mice. THP1 and BMDMs were cultured at 37 °C and 5% CO_2_ in DMEM media (Corning Cellgro) containing FBS (10%, Corning Cellgro) and penicillin/streptomycin (1%, Corning Cellgro). THP1 monocytes were treated with PMA (phorbol 12-myristate 13-acetate; 50 ng/mL, Sigma) for 3 days to differentiate into THP1 macrophages. BMDMs were differentiated with M-CSF (macrophage colony-stimulating factor; 20 ng/mL, Sigma) for 5 days.

### 2.3. Conditioned Medium (CM) Collection

Cells were seeded at the following optimized densities: human immortalized NF1^−/−^ Schwann cells (5 × 10^5^/mL), primary murine Nf1^−/−^ DNSCs (5 × 10^5^/mL), and human THP1 cells (1 × 10^6^/mL) in DMEM media with 1% FBS. After 24 h, the media was collected, centrifuged at 1500 rpm for 10 min, and the supernatant was removed for experimental treatment.

### 2.4. Cytokine Production

Culture supernatants from BMDMs treated with 50% CM from primary murine Nf1^−/−^ DNSCs for 24 h were collected and analyzed using the ProcartaPlex™ Mouse Immune Monitoring Panel (48plex, EPX480-20834-901, Thermo Fisher Scientific, Grand Island, NY, USA), following the manufacture protocol. Samples were measured by Luminex 200 (Luminex Corporation, Austin, TX, USA), and data analysis was performed using the ProcartaPlex Analysis App (Thermo Fisher Scientific). To account for cytokines present in the CM, parallel control CM from Nf1^−/−^ DNSCs were incubated without BMDMs for the same duration and analyzed. Baseline cytokine levels were subtracted from the CM samples that had been incubated with BMDMs, and data were further normalized to CM-untreated BMDMs (treated with standard media) to ensure changes reflected macrophage activation.

Culture supernatants from human THP1 cells treated with 50% CM from human immortalized NF1^−/−^ Schwann cells for 24 h were collected and analyzed using human IL-6 ELISA Kit (KHC0061, Thermo Fisher Scientific), human TNFα ELISA Kit (KAC1751, Thermo Fisher Scientific), and human CCL2 (MCP-1) ELISA Kit (BMS281, Thermo Fisher Scientific), following the manufacture protocol. Samples were measured using a VersaMax Microplate Reader (Molecular Devices, San Jose, CA, USA). Absorbance was read at 450 nm, and data analysis was performed using SoftMax Pro 5.4.1 software (Molecular Devices). For these experiments, control CM incubated without THP1 cells was similarly processed and subtracted to account for baseline cytokine levels.

### 2.5. Western Blot

Treated cells were lysed in RIPA buffer (1 M Tris-Cl, pH 7.4, 0.5 M EDTA, 5 M NaCl, 1% triton-X, 25 mM sodium deoxycholate, 0.1% SDS) with protease inhibitors (1% PMSF, 0.5% apoprotein, 0.1% leupeptin). xTractor Buffer (Takara Bio, San Jose, CA, USA) was used to extract high-molecular-weight proteins, such as NF1, using the same protease inhibitors described above. Lysis buffer was added to each sample, followed by sonication and vortexing three times and centrifugation for 10 min at 13,000 rpm. Protein concentrations of the supernatants were determined using a BCA assay (Sigma). Isolated proteins were fractionated using 10% SDS-PAGE gels or 4–20% precast polyacrylamide gels (Bio-Rad, Hercules, CA, USA) and transferred to polyvinylidene difluoride (PVDF) membranes. Membranes were incubated with pERK (RRID:AB_2315112), ERK (RRID:AB_330744), and vinculin (RRID:AB_2728768) (1:1000, all from Cell Signaling, Danvers, MA, USA), NF1 (1:1000, Abcam, Cambridge, MA, USA, RRID:AB_444142), and GAPDH (1:2000, Santa Cruz, Dallas, TX, USA, RRID:AB_627678) primary antibodies. Following incubation with the appropriate anti-rabbit or anti-mouse secondary antibodies conjugated to HRP (Cell Signaling), a signal was detected using the ECL Western Blotting Substrate (GE Healthcare, Chicago, IL, USA), and protein levels were quantified using ImageJ 5.2 (NIH, RRID:SCR_003070) and statistically analyzed using GraphPad Prism v10 (RRID:SCR_002798).

### 2.6. Cell Viability

Human immortalized NF1^−/−^ Schwann cells and primary murine Nf1^−/−^ DNSCs were plated at a density of 1500 cells per well in 96-well plates and allowed to adhere overnight. The following day, cells were treated with increasing concentrations of drugs (0–1000 nM selumetinib, 0–2000 nM MSU-42011, or the combination) or 200 nM MSU-42011, 50 nM selumetinib, and the combination with or without 50% CM from human THP1 cells. At 72 h post-treatment, 50 μL MTT (3-[4,5-dimethylthiazol-2-yl]-2,5-diphenyltetrazolium bromide; Sigma) was added, and plates were incubated at 37 °C for approximately 4 h. Supernatant was then removed, and 100 μL developing solution (0.04 N HCl in isopropanol) was added. Samples were measured by a VersaMax Microplate Reader (Molecular Devices). Absorbance was read at 570 nm, and data analysis was performed using SoftMax Pro 5.4.1 software (Molecular Devices).

### 2.7. Real Time-PCR

Flash-frozen tumors were processed for total RNA extraction using the RNeasy Mini Kit (Qiagen, Germantown, MD, USA), and RNA from treated cells was isolated with TRIzol (Invitrogen, Waltham, MA, USA), both per the manufacturer’s instructions. RNA purity and quality were assessed by Nanodrop. Two micrograms of RNA were reverse transcribed to cDNA with the High-Capacity cDNA Reverse Transcription Kit (Thermo Fisher Scientific), following optimized thermal cycling conditions (10 min at 25 °C, 2 h at 37 °C, 5 min at 85 °C, and storage at 4 °C). For real-time PCR, the Fast SYBR™ Green Master Mix (Thermo Fisher Scientific) was used with template cDNA and optimized forward and reverse primers ordered from IDT (Supplemental Table S1). The QuantStudio 7 Flex Real-Time PCR system (Thermo Fisher Scientific) was used with the optimized qPCR conditions (20 s at 95 °C, followed by 40 cycles of 1 s at 95 °C and 20 s at 60 °C, with a final step of 1 s at 95 °C, 20 s at 60 °C, and 1 s at 95 °C). Fold change in mRNA expression was quantified using the ^ΔΔ^Ct method, normalized to the control as indicated in the respective figure legends.

### 2.8. In Vivo Experiments

Murine LL2 lung cancer cells (1 × 10^6^ cells/mouse) or murine Nf1-related MPNST cells (3 × 10^5^ cells/mouse) were injected into the flank of male and female C57BL/6 mice. Mice were age-matched (7–8 weeks old, average weight 20–25 g) and excluded if they exhibited signs of illness or abnormal physiology before tumor implantation. Once the tumors reached 3–4 mm in diameter, mice were randomized into groups and treated as indicated in the respective figure legends. Dosing solutions for treatment were prepared on the first day of dosing. The test substances were dissolved in a mixture of DMSO (10%), Cremophor (10%), and PBS (80%) and administered via intraperitoneal injection. The required volume of vehicle or drug solution for each mouse was calculated based on the most recent individual body weight. Tumors were measured by calipers twice per week (LL2) or every two days (MPNST), and body weight was measured once per week to monitor tumor growth and toxicity during the treatment period. At the time of necropsy, mice were euthanized using carbon dioxide. Tumors were harvested, weighed, and divided into two pieces. One piece was fixed in formalin for immunohistochemical analysis, while the other was flash-frozen and stored for RT-PCR. Blood samples were collected by cardiac puncture into tubes containing heparin as an anticoagulant and centrifuged at 5000 rpm for 5 min to separate plasma for triglyceride assay. To minimize bias, investigators were blinded to the treatment groups during data collection and analysis.

### 2.9. Immunohistochemistry

Harvested tumors were fixed in 10% phosphate-buffered formalin for at least 48 h, embedded in paraffin blocks, and sectioned. Endogenous peroxidase activity was quenched using hydrogen peroxide. Sections were then immunostained with antibodies against CD206 (1:200, Abcam, RRID:AB_1523910), FOXP3 (1:50, Cell Signaling, RRID:AB_2797979), and pERK (1:50, Cell Signaling, RRID:AB_2315112) and visualized with biotinylated anti-rabbit secondary antibodies (Cell Signaling). A signal was detected using a DAB substrate (Cell Signaling), following the manufacturer’s recommendations. Sections were counterstained with hematoxylin (Vector Labs, Burlingame, CA, USA). The positive staining signals were quantified using ImageJ 5.2 (NIH, RRID:SCR_003070), and the data were statistically analyzed with GraphPad Prism v10 (RRID:SCR_002798).

### 2.10. Flow Cytometry

Flank tumors and spleens were harvested from the MPNST mouse model treated as described in the ‘In Vivo Experiments’ section above. Samples were dissociated using the gentleMACS™ Dissociator (Miltenyi Biotec, Gaithersburg, MD, USA) with the 37C_m_TDK_1 program and a digestion medium containing collagenase (300 U/mL, Sigma), dispase (1 U/mL, Worthington, Lakewood, NJ, USA), and DNAse (2 U/mL, Calbiochem, Burlington, MA, USA). Cells were passed through a 40 µm cell strainer (BD Falcon, Glendale, AZ, USA), and red blood cells were removed using a lysis solution. The resulting single cells were resuspended in Brilliant Buffer (BD Biosciences, Sparks Glencoe, MD, USA) and stained at 4 °C for 30 min with the following antibodies: CD4-BV750 (1:100, Biolegend, San Diego, CA, USA, GK1.5), CD25-PE (0.5:100, Biolegend, 3C7), CD8-BV605 (1:100, Biolegend, 104732), FOXP3-PE-Cy5 (1:100, eBioscience, FJK-16s), CD206-BV421 (1:100, Biolegend, C068C2), F4/80-APC-Fire 750 (1:100, Biolegend, BM8), IA-IE-BV650 (0.5:100, Biolegend, M5/114.15.2), CD163-APC (1:100, Biolegend, S15049F), CD45-PerCP-Cy5.5 (0.5:100, Biolegend, 30-F11), CD11b-FITC (1:100, Miltenyi Biotec, M1/70.15.11.5), CD11c-BV510 (1:100, Biolegend, M1/70), CD19-BV570 (0.5:100, Biolegend, 6D5), CD3-Vioblue (5:100, Miltenyi Biotec, 17A2), Viability–LIVE-DEAD Far Red (0.5:100, Thermo Fisher Scientific), and 5 μg/mL anti-mouse CD16/CD32 antibody (Biolegend) to reduce antibody binding to Fc receptors. Cells were analyzed using Cytek Aurora and FlowJo v10.0.7r2 software (Tree Star). The gating strategy used is shown in Figure S2.

### 2.11. Triglyceride Assay

Triglyceride levels in plasma were quantified using the Triglyceride Quantification Assay Kit (Abcam, ab65336), following the recommended protocol supplied by the manufacturer for colorimetric readout.

### 2.12. Statistical Analysis

GraphPad Prism v10 (RRID:SCR_002798) was used for all data analysis. Data are presented as the mean ± standard deviation or the mean ± standard error as indicated in the respective figure legends. The in vitro and ex vivo experiments were performed in triplicate for each treatment (cell viability and real-time PCR), and independent experiments were repeated at least three times. Data from in vitro and ex vivo experiments followed normal distributions and were analyzed using one-way ANOVA, with significant differences between groups assessed by the Tukey HSD multiple comparison method. For in vivo experiments, data were analyzed using SigmaStat 3.5, applying one-way ANOVA followed by the Holm–Sidak test for multiple comparisons when the data were normally distributed. For non-normally distributed data, the Kruskal–Wallis one-way ANOVA on ranks was used, followed by the Dunn test for multiple comparisons. Statistical significance was defined as *p* < 0.05 for all analyses.

## 3. Results

### 3.1. MSU-42011 Reduced Tumor Burden, pERK Levels, and Tumor-Promoting CD206^+^ Macrophages in a Subcutaneous Kras-Driven Lung Cancer Model

**Figure 1:**
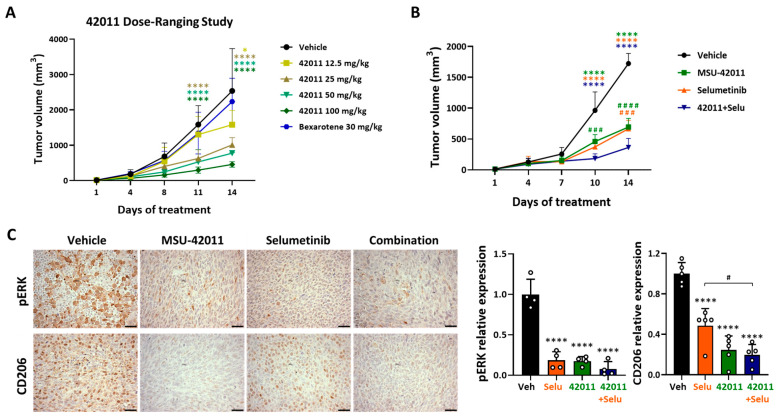
MSU-42011, alone or in combination with selumetinib, suppressed tumor growth and reduced pERK and CD206^+^ levels in an immunocompetent LL2 model of lung cancer. Mouse LL2 lung cancer cells were injected into the flank of male C57BL/6 mice. Once the tumors reached 3–4 mm in diameter, mice were treated i.p. once per day, 5 days per week for 14 days with (**A**) vehicle, MSU-42011 (12.5–100 mg/kg), or bexarotene (30 mg/kg) or with (**B**,**C**) vehicle, 25 mg/kg MSU-42011 (42011), 10 mg/kg selumetinib (Selu), or the combination. (**A**,**B**) Tumor volumes were measured by calipers twice per week. Data represent means ± standard deviations (*n* = 7–9). * *p* < 0.05, **** *p* < 0.0001 vs. treated group; ^###^ *p* < 0.001, ^####^ *p* < 0.0001 vs. combination. (**C**) Tumors were harvested at the time of necropsy for immunohistochemical detection of pERK and CD206. Data represent means ± standard deviations (*n* = 4–5). Scale bar = 60 microns. **** *p* < 0.0001 vs. vehicle; ^#^ *p* < 0.05.

In our previously published studies, the RXR agonist MSU-42011 can prevent or treat carcinogenesis in a Kras-driven lung cancer model [29,30]. In this model, the anti-tumor effects of MSU-42011 are due to its ability to reduce tumor-promoting CD206^+^ macrophages and FOXP3^+^ Tregs and increase tumor-killing cells [29,30]. Moreover, MSU-42011 reduces the level of pERK in both lung and mammary murine tumors [29]. Therefore, to test if MSU-42011 can enhance the effects of a clinically approved MEK inhibitor, selumetinib [15], we implanted murine LL2 lung cancer cells, a *Kras/Nras* mutant cell line [36], into the flank of C57BL/6 mice (1 × 10^6^ cells/mouse). Once the tumors reached 3–4 mm in diameter, mice were randomized into groups and treated intraperitoneally (i.p.) once per day, five days per week, for 14 days with a vehicle, MSU-42011 (12.5–100 mg/kg or 25 mg/kg), selumetinib (10 mg/kg) [42], or the combination. The FDA-approved synthetic RXR agonist, bexarotene (Targretin^(R)^, 30 mg/kg), used for treating cutaneous T-cell lymphoma [43], was also evaluated. Tumors were measured twice per week. MSU-42011, but not bexarotene, significantly (on day 11, 25–100 mg/kg, *p* < 0.0001; on day 14, 12.5 mg/kg, *p* = 0.023 and 25–100 mg/kg, *p* < 0.0001) reduced tumor volume in a dose-dependent manner compared to the vehicle treatment (Figure 1A). Increasing concentrations of MSU-42011 were well tolerated, did not cause body weight loss (Figure S3A), and did not increase plasma triglycerides (Figure S3B). An increase in triglycerides was observed with bexarotene (Figure S3B), as previously reported [44].

The 25 mg/kg dose provided significant biological effects without elevating triglyceride levels, and it was the optimal dose where we saw a strong therapeutic response for combination therapy [29,30], making it suitable for subsequent studies. Treatment with 25 mg/kg MSU-42011 (458 mm^3^, *p* < 0.0001) or 10 mg/kg selumetinib (373 mm^3^, *p* < 0.0001), and the combination (182 mm^3^, *p* < 0.0001), significantly reduced tumor volume after 10 days compared to the vehicle (964 mm^3^) (Figure 1B). Moreover, by day 10, the combination treatment showed a 60.3% greater inhibition of tumor volume compared to MSU-42011 alone (MSU-42011, 458 mm^3^ vs. combination, 182 mm^3^, *p* = 0.0003) but not with selumetinib. By day 14, at the end of treatment, the combination significantly reduced tumor volume by 47.8% compared to MSU-42011 alone and by 45.5% compared to selumetinib alone (MSU-42011, 695 mm^3^, *p* < 0.0001; selumetinib, 666 mm^3^, *p* = 0.0007 vs. combination, 363 mm^3^) (Figure 1B). No differences were observed in body weight between the treatment groups (Figure S3C). After 14 days of treatment, tumors were harvested for immunohistochemical (IHC) staining to assess the levels of pERK and CD206 (tumor-promoting macrophage marker). Both selumetinib and MSU-42011 alone significantly reduced pERK levels by 81.44% (*p* < 0.0001) and 82.51% (*p* < 0.0001), respectively, and decreased the number of CD206^+^ macrophages by 51.46% (*p* < 0.0001) and 75.41% (*p* < 0.0001) compared to vehicle treatment. The combination treatment resulted in a greater reduction, lowering pERK levels to 92.13% (*p* < 0.0001) and CD206^+^ macrophages to 78.35% (*p* < 0.0001) compared to vehicle treatment within the tumors. However, the difference relative to single-agent treatments was modest, with only the selumetinib-treated group showing a significant difference in CD206^+^ macrophages when compared to the combination treatment (Figure 1C).

### 3.2. MSU-42011, Selumetinib, and the Combination Suppressed Tumor Growth, Reduced pERK Levels, and Modulated the Tumor Microenvironment in a Mouse Model of MPNST

**Figure 2:**
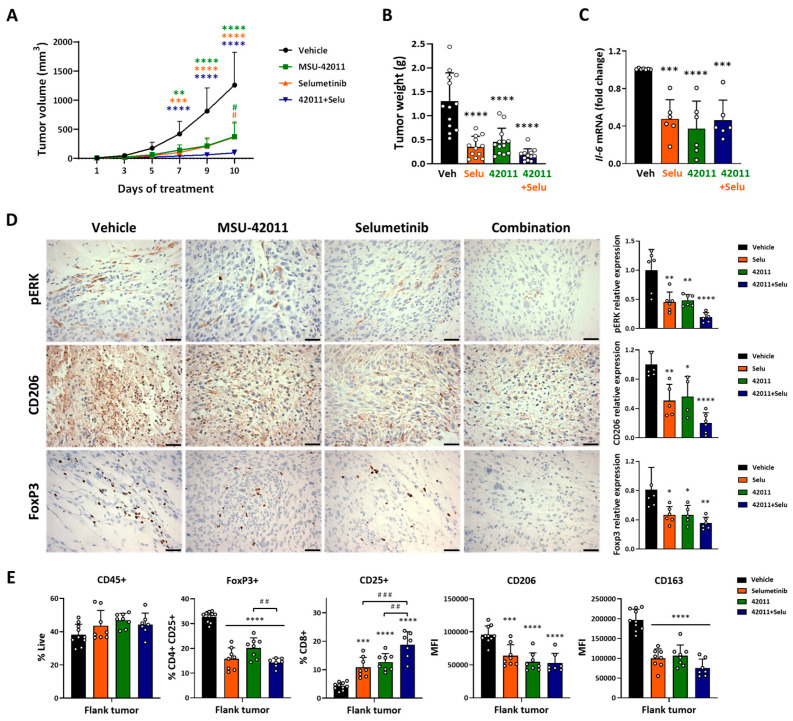
MSU-42011, selumetinib, and the combination reduced tumor growth, pERK levels, tumor-promoting macrophages, FOXP3^+^ Treg, and *Il6* mRNA expression, while increasing activated CD8^+^ T cells in an immunocompetent mouse model of MPNST. Mouse Nf1-related MPNST cells (mMPNST) were injected into the flank of both male and female C57BL/6 mice. Once the tumors reached 3–4 mm in diameter, mice were treated i.p. once per day for 10 days with vehicle, 25 mg/kg MSU-42011, 10 mg/kg selumetinib, or the combination. (**A**) Tumor volumes were measured by calipers every two days. Data represent means ± standard deviations (*n* = 12–13). ** *p* < 0.01, *** *p* < 0.001, **** *p* < 0.0001 vs. treated group; ^#^ *p* < 0.05 vs. combination. (**B**) Tumor weights were recorded at the time of necropsy (*n* = 12–13). (**C**) Tumors were harvested for *Il6* mRNA expression analysis by qPCR (*n* = 6) (**D**) for immunohistochemical detection of pERK, CD206, and FOXP3 (*n* = 6). Scale bar = 60 microns or (**E**) for flow cytometry analysis of immune cell populations using whole tumor lysates (*n* = 8–10). Data represent means ± standard deviations. * *p* < 0.05, ** *p* < 0.01, *** *p* < 0.001, **** *p* < 0.0001 vs. vehicle; ^##^ *p* < 0.01, ^###^ *p* < 0.001.

Due to the importance of immune cells in the progression of neurofibromas [24,25] and the in vivo efficacy of MSU-42011 in Kras-driven lung cancer (Figure 1) [29,30], which shares similarities with NF1 models, immunocompetent C57BL/6 mice were used to evaluate the immunomodulatory and anti-tumor effects of MSU-42011 in vivo in MPNST. Selumetinib has been tested in clinical trials for MPNST treatment, primarily focusing on developing effective combination strategies with the mTOR inhibitor sirolimus (NCT03433183) or the bromodomain inhibitor AZD5153 plus the programmed death-ligand 1 (PD-L1) antibody durvalumab (NCT05253131) [45]. Murine Nf1-related MPNST cells, which harbor both *Nf1* and *Tp53* mutations that enhance aggressiveness and promote tumor formation [39] were injected into the flank of C57BL/6 mice (3 × 10^5^ cells/mouse). Due to the high aggressiveness of this cell, a continuous 10-day treatment regimen was used to prevent ulceration from treatment gaps. Once the tumors reached 3–4 mm in diameter, the mice were treated i.p. once daily for 10 days with the vehicle, 25 mg/kg MSU-42011, 10 mg/kg selumetinib, or the combination. Tumor growth was monitored every two days during the treatment period. MSU-42011 (139 mm^3^, *p* = 0.0072) and selumetinib (99 mm^3^, *p* = 0.0009), either alone or in combination (40 mm^3^, *p* < 0.0001), significantly reduced tumor volume after 7 days compared to the vehicle treatment (419 mm^3^) (Figure 2A). At the 10-day end point, the combination treatment showed a greater reduction in tumor volume compared to each single-agent treatment (vehicle, 1260 mm^3^, *p* < 0.0001; MSU-42011, 377 mm^3^, *p* = 0.0115; selumetinib, 365 mm^3^, *p* = 0.0277 vs. combination, 95 mm^3^). Additionally, at the 10-day end point, tumor weights were also significantly reduced compared to vehicle treatment (MSU-42011, 0.46 g; selumetinib, 0.35 g; combination, 0.18 g vs. vehicle, 1.30 g, *p* < 0.0001) (Figure 2B). No differences in body weight were observed among the groups (Figure S4A), similar to what we observed in the LL2 model (Figure S3C). In contrast, bexarotene, the FDA-approved RXR agonist, in the same mouse model of MPNST, showed no effects on tumor burden (Figure S5).

MEK inhibitors have been shown to decrease long-chain fatty acids, reducing lipid oxidation, which was accompanied by an increase in triacylglycerides in some patients with NF1-associated PNF [46]. In the LL2 lung cancer model, we observed higher plasma triglyceride levels in the selumetinib-treated group but not in the MSU-42011-treated group (Figure S3D). In the mouse model of MPNST, selumetinib and bexarotene increased plasma levels of triglycerides, but no increase was observed in MSU-42011-treated mice (Figure S4B,C). The increase observed in the combination treatment may be due to selumetinib (Figures S3D and S4B). These data point out that MSU-42011, unlike selumetinib and bexarotene, did not induce triglyceride accumulation, indicating a potentially safer metabolic profile for use in cancer treatment.

Schwannomatosis (SWN), a distinct NF-related disorder, often causes severe chronic pain leading to disability [47,48]. Studies in SWN cell lines show that macrophages trigger pain responses through IL-6 overproduction, with a fourteen-fold increase in secretion observed in mouse peritoneal macrophages stimulated by SWN-conditioned medium [49]. Moreover, IL-6 blockade significantly reduces pain in orthotopic sciatic nerve and spine patient-derived xenograft (PDX) models [49]. Notably, both selumetinib and MSU-42011, alone and in combination, reduced *Il-6* mRNA expression by 52.76% (*p* = 0.0007), 63.12% (*p* < 0.0001), and 54.26% (*p* = 0.0005) compared with vehicle treatment, as measured in tumor lysates (Figure 2C). Since IL-6 overproduction in NF1-deficient models is mostly associated with increased infiltration and activation of myeloid-derived suppressor cells (MDSCs) [22], and previous observations that MSU-42011 reduced expression of CD206 tumor-promoting macrophage marker (Figure 1C) [29,30], we analyzed the levels of CD206 in MPNST flank tumors by IHC staining. Selumetinib and MSU-42011 reduced CD206 expression by approximately 49% (*p* = 0.0043) and 44% (*p* = 0.0172), respectively; the combination was greatly effective, with an 80% (*p* < 0.0001) reduction compared with vehicle treatment (Figure 2D). High numbers of FOXP3^+^ Tregs in tumors are generally associated with worse overall survival in breast cancer [50]. As observed in the HER2^+^ breast cancer and Kras-driven lung cancer models [29,30], MSU-42011 (~43%, *p* = 0.0246) and selumetinib (~43%, *p* = 0.0175) alone or in combination (~57%, *p* = 0.0016) reduced the expression of FOXP3^+^ in the flank tumors of MPNST compared with vehicle treatment. Moreover, despite not carrying an *RAS* mutation, MSU-42011 (~52%, *p* = 0.0018), selumetinib (~55%, *p* = 0.0010), and the combination (~81%, *p* < 0.0001) reduced the expression of pERK compared with vehicle treatment (Figure 2D). To complement IHC analysis, we performed flow cytometry on whole tumor lysates to further characterize the immune landscape of MPNST tumors. Drug treatments did not affect the total CD45^+^ immune cell population (Figure 2E). Selumetinib, MSU-42011, and the combination significantly reduced the percentage of FOXP3^+^CD25^+^CD4^+^ Treg (~52%; ~38%; ~56% vs. vehicle, *p* < 0.0001) and decreased the mean fluorescence intensity (MFI) of CD206^+^ (~33%, *p* = 0.0004; ~43%, *p* < 0.0001; ~45%, *p* < 0.0001 vs. vehicle) and CD163^+^ tumor-promoting macrophages (~49%; ~46%; ~62% vs. vehicle, *p* < 0.0001) (Figure 2E), in agreement with the reduction in FOXP3 and CD206 expressions observed by IHC analysis (Figure 2D). Furthermore, selumetinib and MSU-42011 increased the percentage of activated CD25^+^CD8^+^ T cells (2.5X, *p* = 0.0007; 3.0X, *p* < 0.0001 vs. vehicle), and the combination enhanced this response to a 4.4-fold increase compared with the vehicle (*p* < 0.0001). No alterations in the percentage or MFI of the aforementioned immune cell populations were observed in the spleen from the same cohort of mice (Figure S6). The combination treatment demonstrated superior efficacy in reducing the tumor burden and modulating the tumor microenvironment, highlighting its potential as a therapeutic strategy for aggressive MPNSTs.

### 3.3. Selumetinib Reduced pERK Levels in NF1-Deficient Tumor Cells, While MSU-42011 Showed Limited Effects In Vitro

To understand the potential mechanisms underlying the anti-tumor effects of MSU-42011 in NF1, we used several mouse and human NF1-deficient cell lines to perform in vitro studies. These included human immortalized NF1^−/−^ Schwann cells [37,38], murine Nf1-related MPNSTs (referred to as mMPNST) [39], and primary murine Nf1 Cdkn2a^−/−^ and Nf1^−/−^ DNSCs (referred to as mSC, Nf1^f/f^ Cre-; mPNF, Nf1^f/f^ Cre+; mMPNST precursor, Nf1^f/f^ Cdkn2a^f/f^ Cre+) [41]. pERK is elevated in these human and mouse cells due to both a germline and somatic mutation in *NF1/Nf1*, which activates RAS and the downstream MAPK signaling pathway [37,39,41].

**Figure 3:**
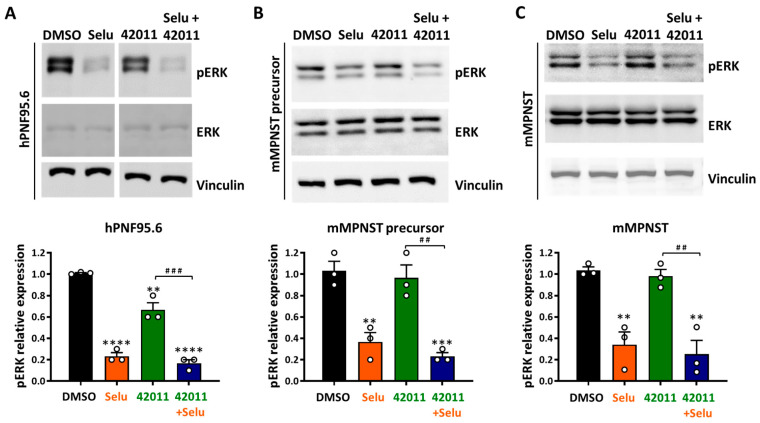
Selumetinib reduced pERK levels in NF1-deficient cells, while MSU-42011 had a limited effect alone and no additive effect in combination. (**A**) ipNF95.6 human PNF cells (hPNF95.6), (**B**) mouse MPNST precursor cells (Nf1^f/f^ Cdkn2a^f/f^ Cre+), and (**C**) mouse Nf1-related MPNST cells (mMPNST) were treated with 50 nM selumetinib (Selu), 200 nM MSU-42011 (42011), or the combination for 3 h. The level of pERK was evaluated by Western blotting. Data represent means ± standard deviations (*n* = 3). ** *p* < 0.01, *** *p* < 0.001, **** *p* < 0.0001 vs. DMSO; ^##^ *p* < 0.01, ^###^ *p* < 0.001. The uncropped blots are shown in File S1.

In human PNF cells (hPNF95.6), treatment with MSU-420011 at 200 nM reduced the levels of pERK starting at 0.5 h, reaching peak reduction at 3 h, but this reduction was lost at 6 and 24 h. In contrast, 50 nM selumetinib reduced pERK levels from 0.5 to 24 h (Figure S7A). Based on the maximum reduction in pERK by MSU-42011 at 3 h, this time point was selected as the optimal treatment time for pERK level analysis. After 3 h of treatment, selumetinib significantly reduced pERK levels in human PNF cells, mouse MPNST precursor cells, and mouse MPNST cells by approximately 77% (*p* < 0.0001), 63% (*p* = 0.0031), and 66% (*p* = 0.0036), respectively, compared with vehicle-treated cells (Figure 3). MSU-42011 alone reduced pERK levels in human PNF cells by about 33% (*p* = 0.0016 vs. vehicle-treated cells) (Figure 3A). MSU-42011 was ineffective in reducing pERK in mouse MPNST precursor cells and mouse MPNST cells (Figure 3B,C). The combination treatment reduced pERK levels by 83% (*p* < 0.0001), 77% (*p* = 0.0009), and 75% (*p* = 0.0017) in human PNF cells, mouse MPNST precursor cells, and mouse MPNST cells but showed no additive effect compared to single-agent treatment (Figure 3). Additionally, human PNF cells and mouse MPNST precursor cells were treated with increasing concentrations of MSU-42011 and selumetinib for 72 h, alone or in combination, and cell viability was measured using the MTT assay. A trend of reduced cell viability was observed with increasing concentrations of the combination in both cell lines, though the reduction in mouse MPNST precursor cells may be primarily attributed to selumetinib. MPNST precursor cells did not respond to MSU-42011 alone (Figure S7B). These findings suggest that the combination of MSU-42011 and selumetinib reduced pERK levels. However, the reduction in viability of NF1-deficient cells is most likely due to selumetinib.

In Nf1-deficient mice, increased macrophage infiltration correlates with disease severity [25], and to mimic the communication between tumor-associated macrophages (TAMs) and tumor cells, conditioned media (CM) from macrophages and NF1-deficient cells were used to model this interaction. CM from macrophages were collected after a 24 h incubation with 1 × 10^6^/mL human THP1 monocytes (CM) or THP1 macrophages (PMA-CM) differentiated with 50 ng/mL PMA for 3 days [51]. The 50% CM were subsequently added to human PNF cells, with treatments administered simultaneously with the vehicle, 200 nM MSU-42011, 50 nM selumetinib, or the combination for either 3 h (to assess pERK levels) or 72 h (to assess cell viability). In a similar study using MCF7 breast cancer cells, CM from macrophages was reported to induce ERK signaling that can be inhibited by MEK inhibitor PD98059 [52]. Contrary to the increase in pERK observed in MCF7 cells, human PNF cells stimulated with CM from both THP1 monocytes or THP1 macrophages showed modestly reduced pERK levels (*p* > 0.05) (Figure S8A). Although MSU-42011 alone had no impact on pERK, its combination with selumetinib in the presence of CM led to a further reduction in pERK levels, an effect likely driven primarily by selumetinib (Figure S8A). Cell viability, assessed using an MTT assay and normalized to control without CM treatment , revealed that CM from THP1 monocytes and macrophages reduced the viability of human PNF cells by approximately 20%. Moreover, the combination treatment further reduced the cell viability (Figure S8B).

### 3.4. NF1-Deficient Cells Increased the Secretion and Expression of Cytokine and Chemokine in Macrophages, and Treatment with MSU-42011 and Selumetinib Partially Reduced the Increase

*NF1* mutations have broad effects on immune cells and cytokine signaling, driven by a hyperactive MAPK signaling pathway [22,53], leading to elevated levels of inflammatory signals such as IL-6, VEGF, and CCL2 in patients with NF1 [22,24,49,54]. To assess the impact of NF1-deficient cells on macrophage activation, CM were collected after a 24 h incubation with 5 × 10^5^/mL murine and human NF1-deficient cells. The collected CM were then added at 50% to murine primary bone marrow-derived macrophages (BMDMs) and human THP1 cells, respectively, with treatments administered simultaneously for 24 h. BMDMs were isolated from C57BL/6 mice and differentiated with 20 ng/mL M-CSF for 5 days [55]. After a 24 h CM stimulation and treatment, the expression and secretion of cytokines and chemokines in macrophages (BMDMs and THP1 cells) were analyzed by RT-PCR, ELISA, and multiplex assay.

**Figure 4:**
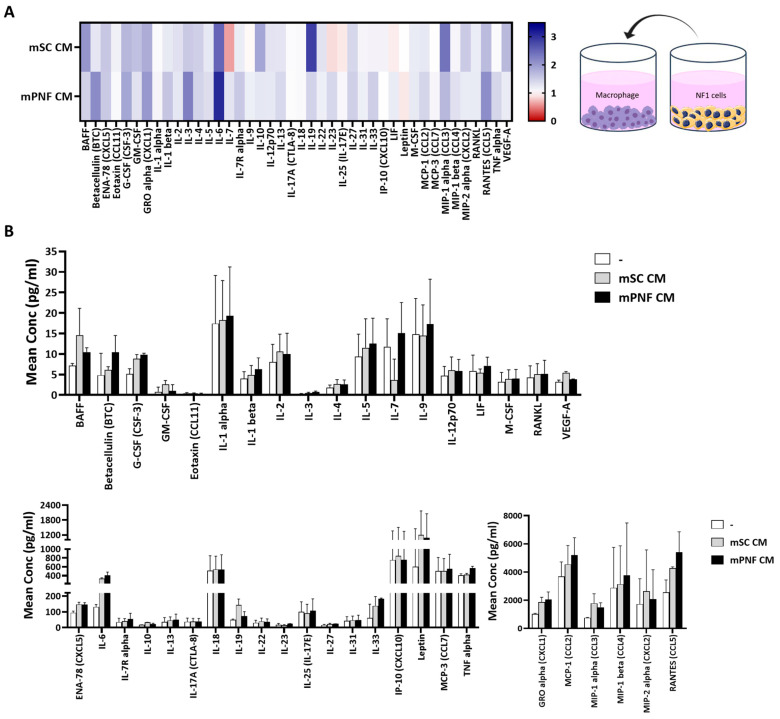
Conditioned media (CM) from murine Schwann cells and PNF cells increased cytokine and chemokine secretion in bone marrow-derived macrophages (BMDMs). Supernatants from BMDMs, differentiated with 20 ng/mL M-CSF for 5 days and then treated with 50% CM from mouse normal Schwann cells (Nf1^f/f^ Cre-) and PNF cells (Nf1^f/f^ Cre+) for 24 h, were analyzed by a multiplex assay. (**A**) The heat map illustrated the relative analyte concentrations, normalized to the BMDMs without CM treatment. (**B**) The mean concentrations were categorized as low (<20 pg/mL), medium (<1400 pg/mL), and high (<5000 pg/mL). Data were averaged across two replicates.

**Figure 5:**
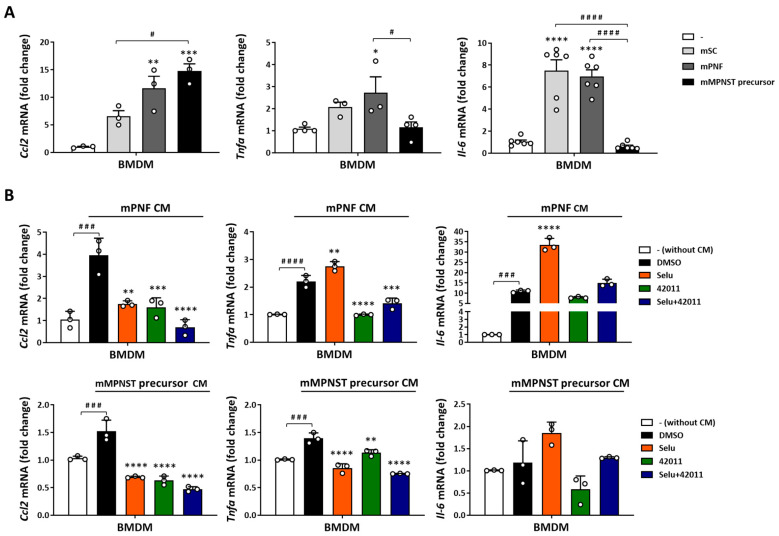
CM from mouse PNF cells and MPNST precursor cells induced cytokine mRNA expression in BMDMs, which was reduced by combination treatment with MSU-42011 and selumetinib. (**A**) BMDMs differentiated with 20 ng/mL M-CSF for 5 days were treated with 50% CM from mouse Schwann cells (Nf1^f/f^ Cre-), PNF cells (Nf1^f/f^ Cre+), and MPNST precursor cells (Nf1^f/f^ Cdkn2a^f/f^ Cre+) for 24 h. Data represent means ± standard deviations (*n* = 3–6). (**B**) Differentiated BMDMs were treated with 50% CM from mouse PNF cells (mPNF CM), MPNST precursor cells (mMPNST precursor CM), and drugs (50 nM selumetinib, 200 nM MSU-42011, or the combination) for 24 h. mRNA expression was evaluated by qPCR and normalized to the BMDMs without CM and drug treatment. Data are representative of three independent experiments. * *p* < 0.05, ** *p* < 0.01, *** *p* < 0.001, **** *p* < 0.0001 vs. BMDMs without CM treatment (**A**) or vs. DMSO (**B**); ^#^ *p* < 0.05, ^###^ *p* < 0.001, ^####^ *p* < 0.0001.

The heatmap from the cytokine/chemokine multiplex assay visually represented the relative concentrations of multiple cytokines and chemokines (Figure 4A). These concentrations were measured in the supernatants of BMDMs treated with CM from both mouse Schwann cells and PNF cells and normalized to BMDMs without CM treatment. CM treatment induced upregulation in the secretion of BAFF, betacellulin (BTC), ENA-78 (CXCL5), G-CSF (CSF-3), GM-CSF, GRO alpha (CXCL1), IL-1 alpha, IL-1 beta, IL-2, IL-3, IL-4, IL-5, IL-6, IL-7R alpha, IL-10, IL-12p70, IL-13, IL-19, IL-22, IL-27, IL-31, IL-33, M-CSF, MCP-1 (CCL2), MIP-1 alpha (CCL3), MIP-1 beta (CCL4), MIP-2 alpha (CXCL2), RANKL, RANTES (CCL5), TNF alpha, and VEGF-A in BMDMs exposed to the CM from both mouse Schwann cells and PNF cells (Figure 4A). Additionally, the bar graph illustrated the concentration levels of the factors in the supernatants of BMDMs exposed to CM from mouse Schwann cells, PNF cells (Figure 4B), and MPNST precursor cells (Figure S9A). Although not statistically significant, CM from PNF cells showed a trend toward increased levels of secretions compared to normal Schwann cells, including betacellulin (BTC), G-CSF (CSF-3), GRO alpha (CXCL1), IL-1 alpha, IL-1 beta, IL-3, IL-5, IL-6, IL-7R alpha, IL-9, IL-13, IL-27, IL-31, IL-33, MCP-1 (CCL2), MIP-1 beta (CCL4), RANTES (CCL5), and TNF alpha (Figure 4B). mRNA expression levels of key cytokines and chemokines were used to confirm the observations made on the multiplex assay. *Ccl2*, *Tnfα*, and *Il-6* mRNA expression increased by approximately 12X (*p* = 0.0026), 2.7X (*p* = 0.0119), and 7X (*p* < 0.0001), respectively, in BMDMs treated with CM from PNF cells compared to non-CM-treated groups. In contrast, MPNST precursor cells also increased *Ccl2* mRNA expression by about 15X (*p* = 0.0005) but did not significantly alter *Tnfα* or *Il-6* levels compared to non-CM-treated groups (Figure 5A). Importantly, the increased *Ccl2* mRNA expression induced by CM from both mouse PNF and MPNST precursor cells was effectively inhibited by 200 nM MSU-42011 (PNF, *p* = 0.0007; MPNST precursor, *p* < 0.0001) and 50 nM selumetinib (PNF, *p* = 0.0012; MPNST precursor, *p* < 0.0001) and further reduced by their combination (PNF and MPNST precursor, *p* < 0.0001) compared to the vehicle treatment. Additionally, the combination treatment reduced *Tnfα* expression in BMDMs exposed to CM from both mouse PNF cells (*p* = 0.0005) and MPNST precursor cells (*p* < 0.0001) (Figure 5B).

Macrophages are broadly categorized as classically activated type 1 (M1) and alternatively activated type 2 (M2) [56,57]. TAMs typically exhibit an M2-like phenotype promoting tumor progression and resistance to chemotherapy [58,59]. While it is well established that many tumors recruit monocytes from circulation and transform them into immunosuppressive M2-like subsets [60], our data showed that mouse PNF cells induced higher expression of both M1 (*iNOS*, 39X, *p* = 0.0026) and M2 (*Arg1*, 12X, *p* = 0.0044) macrophage markers compared to non-CM-treated groups. In contrast, mouse MPNST precursor cells only induced *Arg1* mRNA expression (9X, *p* = 0.0374) in BMDMs, without affecting *iNOS* expression (Figure S9B).

**Figure 6:**
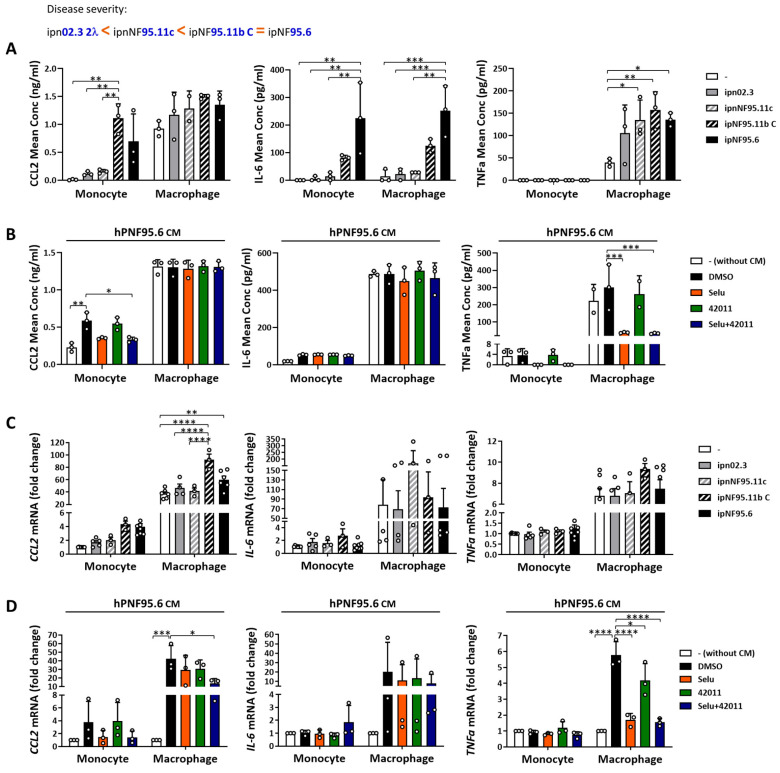
CM from human PNF cells increased cytokine secretion and mRNA expression in THP1 cells, which was reduced by combination treatment with MSU-42011 and selumetinib. THP1 monocytes or macrophages (THP1 cells differentiated with 50 ng/mL PMA for 3 days) were treated with (**A**,**C**) 50% CM from human Schwann cells (ipn02.3 2λ), non-tumor Schwann cells with an *NF1* mutation (ipnNF95.11c), and PNF cells (ipNF95.11bC and ipNF95.6) or with (**B**,**D**) 50% CM from ipNF95.6 human PNF cells (hPNF95.6 CM) and drugs (50 nM selumetinib, 200 nM MSU-42011, or the combination) for 24 h. (**A**,**B**) IL-6, TNFα, and CCL2 secretion in the supernatants were measured by ELISAs. (**C**,**D**) mRNA expression was evaluated by qPCR and normalized to the monocyte without CM treatment. Data represent means ± standard deviations (*n* = 3–8). * *p* < 0.05, ** *p* < 0.01, *** *p* < 0.001, **** *p* < 0.0001.

The results observed in murine cells were also confirmed in human NF1-deficient cells and human THP1 monocytes or THP1 macrophages differentiated as described above. CM from PNF cells (ipNF95.11bC) increased CCL2 secretion in THP1 monocytes (*p* = 0.0019) compared to non-CM-treated groups. Additionally, CM from PNF cells (ipNF95.6) elevated IL-6 secretion in both THP1 monocytes (*p* = 0.0011) and macrophages (*p* = 0.0006), and CM from NF1-mutant non-tumor Schwann cells (ipnNF95.11c, *p* = 0.0196) and PNF cells (ipNF95.11bC, *p* = 0.0026; ipNF95.6, *p* = 0.0431) induced TNFα secretion in THP1 macrophages compared to non-CM-treated groups (Figure 6A). Furthermore, *CCL2* mRNA expression was upregulated 1.5-2.5X in THP1 macrophages treated with CM from PNF cells (ipNF95.11bC, *p* < 0.0001; ipNF95.6, *p* = 0.0018) compared to non-CM-treated groups (Figure 6C). The combination treatment reduced the secretion of CCL2 and TNFα by about 43% (*p* = 0.0288 vs. DMSO) and 89% (*p* = 0.0004 vs. DMSO) in THP1 monocytes and macrophages, respectively (Figure 6B), as well as the mRNA expression of *CCL2* and *TNFα* by about 68% (*p* = 0.0121 vs. DMSO) and 73% (*p* < 0.0001 vs. DMSO) in THP1 macrophages stimulated with CM from PNF cells (Figure 6D). The reductions were also observed in TNFα secretion by selumetinib alone (~87%, *p* = 0.0006 vs. DMSO) (Figure 6B) and in *TNFα* expression by MSU-42011 (~27%, *p* = 0.0165 vs. DMSO) and selumetinib alone (~70%, *p* < 0.0001 vs. DMSO) (Figure 6D) in THP1 macrophages stimulated with CM from PNF cells. Though selumetinib may be the main driver, only the combination treatment reached statistical significance in inhibiting CCL2 protein and mRNA levels. These findings support the hypothesis that NF1 deficiency enhances the activation of inflammatory pathways by increasing both protein and mRNA levels of cytokines and chemokines. Additionally, the combination of MSU-42011 and selumetinib partially reversed these effects, highlighting its potential as a therapeutic intervention.

## 4. Discussions

In this study, we tested the potential of MSU-42011, alone or in combination with selumetinib, to reduce tumor growth in neurofibromatosis, and evaluated the mechanistic effects of this combination. The RAS signaling pathway is constitutively activated in both the LL2 lung cancer model, through an activating *RAS* mutation [36], and the MPNST model, by the loss of *NF1* [2,3,4]. As a result, ERK phosphorylation is highly expressed in both models. MSU-42011 reduced the levels of pERK in both murine models, alone or in combination with selumetinib (Figure 1C and Figure 2D). Targeting RAS or its downstream effectors, such as the MAPK signaling pathway, has been a longstanding goal in cancer research, and recent progress has been made with the development of MAPK and KRAS G12C small-molecule inhibitors [61,62]. However, MPNST cells, in particular, exhibit low responsiveness to MEK inhibition [42], and clinical applicability is limited due to toxicity and resistance [63]. Interestingly, pERK reduction by MSU-42011 was observed exclusively in in vivo models (Figure 1C and Figure 2D). In vitro, the effects of MSU-42011 on pERK reduction and cell viability were limited compared to selumetinib (Figure 3 and Figure S7). This suggests that the primary effects of MSU-42011 may not directly target tumor cells but instead involve alternative mechanisms, such as modulating the TME. Supporting the hypothesis that MSU-42011 modulated inflammatory mediators and immune cell populations in tumors with *KRAS* mutations (Figure 1) or RAS signaling pathway activation (Figure 2). Specifically, the IHC staining showed the ability of MSU-42011 to reduce the expression of CD206 and FOXP3 (Figure 2D), and this was confirmed by flow cytometry, which showed that MSU-42011 reduced tumor-promoting macrophages (low CD206 and CD163), FOXP3^+^ Treg, and increased the presence of activated CD8^+^ T cells in the flank tumors of MPNST (Figure 2E). The immune-modulating properties of MSU-42011 were also evident by using conditioned media (CM) from NF1-deficient cells in BMDMs or THP1 cells, where it reduced the expression and secretion of pro-inflammatory cytokines and chemokines (Figure 5 and Figure 6). Moreover, MSU-42011 showed no anti-tumor efficacy in an athymic A549 lung cancer xenograft mode, further validating the dependency on an intact immune system [29]. This creates a limitation for using MSU-42011 as a standalone therapy in such models, and its application may need to be considered alongside other strategies or in models with an intact immune response.

The RXR agonist MSU-42011 has been shown to reduce FOXP3^+^ Treg and increase activated cytotoxic T cells in several tumor murine models [29], including in MPNST (Figure 2D,E). While the role of T cells in NF1 is poorly understood, their distribution across different tumor stages suggests a dynamic involvement in disease progression. Activated and effector T cells are elevated in patients with a low PNF tumor burden compared to those without PNFs, but these levels decline as the tumor burden increases [64], suggesting a muted T-cell response with a greater tumor burden. Moreover, ANNUBP tumors show enriched immune surveillance and infiltration by CD4^+^FOXP3^−^ and CD8^+^FOXP3^−^ T cells. However, these T cells are significantly diminished in MPNSTs and are replaced by an increase in FOXP3^+^ Tregs [65]. PD-L1, a marker of immune exhaustion, is also significantly enriched in MPNST [66]. Thus, the role of MSU-42011 in reducing FOXP3^+^ Tregs and increasing activated CD8^+^ T cells in the MPNST model is particularly intriguing (Figure 2D,E), potentially alleviating immune suppression and enhancing anti-tumor immunity. These immunological changes by MSU-42011 are highly beneficial for cancer treatment but were not observed with bexarotene [35]. Given the enrichment of PD-L1 in MPNSTs and the ability of MSU-42011 to modulate the immune landscape, combining MSU-42011 with immune checkpoint inhibitors such as anti-PD-1 or anti-PD-L1 may further enhance anti-tumor immunity. Further characterization of the T cell population infiltrating into the flank tumors of MPNST and the role of MSU-42011 in modulating these populations is warranted and currently underway at our laboratory.

Macrophages are one of the dominant immune cell populations in neurofibromas and have long been hypothesized to contribute to neurofibromas development [24]. Studies in mouse models of PNF indicated that neurofibroma-associated macrophages have both anti-tumor and pro-tumor roles at different disease stages, initially inhibiting PNF development and later promoting the growth of established PNF [25]. However, the relative contributions of macrophages to neurofibromas, and specific macrophage functions or subtypes, remain unclear. TAMs typically exhibit an M2-like phenotype that supports tumor progression [58,59]. Yet, in the Nf1^fl/fl^; DhhCre mouse model, macrophages exhibited mixed M1/M2 profiles, expressing M1 markers without clear M2 sub-population clustering in benign neurofibromas [67]. These observations align with our findings that CM from mouse PNF cells induced both M1 (*iNOS*) and M2 (*Arg1*) marker expression in macrophages, while more aggressive tumors, like MPNST precursor cells, skewed macrophages toward an M2-like phenotype, characterized by the increase in only *Arg1* expression (Figure S9B). This mix phenotype is also consistent with the cytokine levels measured by RT-PCR in this report, as mouse PNF cells induced higher pro-inflammatory *Tnfα* or *Il-6* RNA expression compared to MPNST precursor cells (Figure 5A). The ability of PNF cells to drive heightened inflammatory responses in macrophages is further supported by Figure 4B, which shows that CM from PNF cells enhanced cytokine and chemokine secretion in BMDMs compared to normal Schwann cells. Additionally, Figure 6A,C indicates a correlation between NF1 deficiency and increased secretion (CCL2, IL-6, and TNFα) and mRNA expression (*CCL2*), progressing from normal Schwann cells to NF1-mutant non-tumor Schwann cells to PNF cells. These data support the hypothesis that neurofibroma-associated macrophages may help sustain the pro-inflammatory state within the benign neurofibroma microenvironment, contributing to prolonged chronic inflammation that is critical for neurofibroma development and disease progression [25,67]. However, as tumors progress, it remains to be determined whether macrophages become increasingly polarized toward an M2-like phenotype or another tumor-promoting subtype to support tumor growth and how pharmacological intervention with MSU-42011 can intercept this progression.

Bexarotene, the FDA-approved RXR agonist, used for the treatment of cutaneous T-cell lymphoma [43], increased survival in a subset of lung cancer patients [68,69,70] but was not approved, likely due to toxicity and limited potency as a single agent [33]. Despite these challenges, these trials support the potential of RXR agonists for cancer treatment. RXR agonists were initially developed to reduce cancer cell proliferation and induce apoptosis or differentiation [71,72]. However, their immune regulatory effects are particularly significant in diseases with strong inflammatory components, such as cancer, neurodegenerative diseases, and viral infections [73,74,75]. RXRs likely exert immune regulatory effects through multiple immune cell types, including macrophages, dendritic cells, and CD8^+^ T cells [34]. RXRα is highly expressed in macrophages and regulates inflammatory responses via PPARγ and LXR signaling. In dendritic cells, RXR agonists enhance antigen presentation and cell survival. RXR activation also promotes CD8^+^ T cell infiltration, while its disruption impairs T cell proliferation, particularly in CD8^+^ subsets [44]. These findings support the involvement of these immune cells as key mediators of RXR-targeted effects. Despite this, compared to bexarotene [76], MSU-42011 has significant advantages: (1) it does not elevate triglycerides (Figures S3 and S4), (2) it is more potent as a single agent (EC50 for RXR activation: 30 nM for MSU-42011 vs. 55 nM for bexarotene) [30], and (3) it has a stronger immune-modulatory profile [29,30,33]. The differences in efficacy observed between MSU-42011 and bexarotene in the MPNST model, including triglyceride levels and tumor burden (Supplemental Table S2), may be attributed to their distinct mechanisms of action. Bexarotene off-target signaling through RAR can lead to various adverse effects, including triglyceride elevation [44]. In contrast, MSU-42011 is specifically selective for RXR, without induction of other nuclear receptors [44].

While our findings support the therapeutic potential of RXR agonists in NF1-associated models, several limitations should be noted. First, NF1 is a complex genetic disorder characterized by diverse mutation combinations, and our model, NF1-deficient cells with *CDKN2A* and *TP53* deletions, represents only a subset of mutation profiles. Thus, the results may not fully capture the heterogeneity observed in patients. Additionally, the rapid tumor growth in syngeneic mouse models fails to recapitulate the natural progression and complexity of the TME, lacking immune-editing stages seen in patients. To address these issues, we are currently testing MSU-42011 in additional NF1 models (e.g., transgenic mice) to evaluate its interactions with specific pathways, immune cells, and cancer progression, providing insights into its mechanisms of action. We are also conducting intensive immune profiling of the tumors using RNA sequencing to assess other immune populations, including not only tumor-promoting immune cells but also mast cells and cytotoxic T cells, given its previously observed role in increasing activated cytotoxic T cells in other models [29,30]. Further studies are also needed to evaluate the translational relevance of these findings. The combination of the present study and future proposed experiments will enable future clinical trials of MSU-42011 as a single agent or in combination with other current or future therapeutic options, such as MEK inhibitors or immunotherapy.

## 5. Conclusions

In summary, we described the efficacy of MSU-42011 in preclinical NF1-deficient models, its ability to reduce pERK levels and tumor-promoting immune cell populations (CD206/CD163 macrophages and FOXP3^+^ Tregs) and increase activated CD8^+^ T cells within the tumor and its potential to further decrease tumor burden when combined with an MEK inhibitor, selumetinib.

## Data Availability

The data generated in this study are available within the article and its supplementary data files.

## Supplementary Materials

The following supporting information can be downloaded at: https://www.mdpi.com/article/10.3390/cancers17121920/s1, Figure S1. Knockdown efficiency of adenovirus-mediated Cre recombinase targeting Nf1 in DRG/nerve root neurosphere cells (DNSCs). Figure S2. Gating strategy used for flow cytometry analysis of myeloid and T cell populations. Figure S3. Triglyceride levels increased in the bexarotene and selumetinib-treated groups, but not in the MSU-42011-treated group in an immunocompetent LL2 model of lung cancer. Figure S4. Triglyceride levels increased in the 10-day bexarotene and selumetinib-treated groups, but not in the MSU-42011-treated group in a mouse model of MPNST. Figure S5. Bexarotene had no effect in an immunocompetent mouse model of MPNST. Figure S6. Immune cell populations in the spleen. Figure S7. Effects of MSU-42011 and selumetinib on pERK levels and cell viability in NF1-deficient cells. Figure S8. Conditioned media (CM) from human THP1 monocytes or macrophages did not alter pERK levels or cell viability in human PNF cells. Figure S9. CM from murine MPNST precursor cells increased cytokine and chemokine secretion, while CM from murine PNF cells enhanced *Arg1* and *iNOS* mRNA expression in bone marrow-derived macrophages (BMDMs). Table S1: Primer sequences used in qPCR experiments. Table S2: Comparison of MSU-42011 and bexarotene in mouse models of MPNST and LL2 lung cancer. File S1: Western blot information.
