# Supplementary material for: The RXR Agonist MSU-42011 Reduces Tumor Burden in a Murine Preclinical NF1-Deficient Model
Source: Cancers (Basel). 2025 Jun 9;17(12):1920. doi: 10.3390/cancers17121920 (PMC12190937; doi:10.3390/cancers17121920)
Supplement: Supplementary file 1 [file cancers-17-01920-s001.zip › cancers-3616244-supplementary.pdf]

Supplemental Materials

The RXR Agonist MSU-42011 Reduces Tumor Burden in a Murine Preclinical NF1-Deficient Model

Pei-Yu Hung, Jessica A. Moerland, Ana S. Leal, Bilal Aleiwi, Edmund Ellsworth, D Wade Clapp, Verena Staedtke, Renyuan Bai, Karen T. Liby

Supplemental Figures:

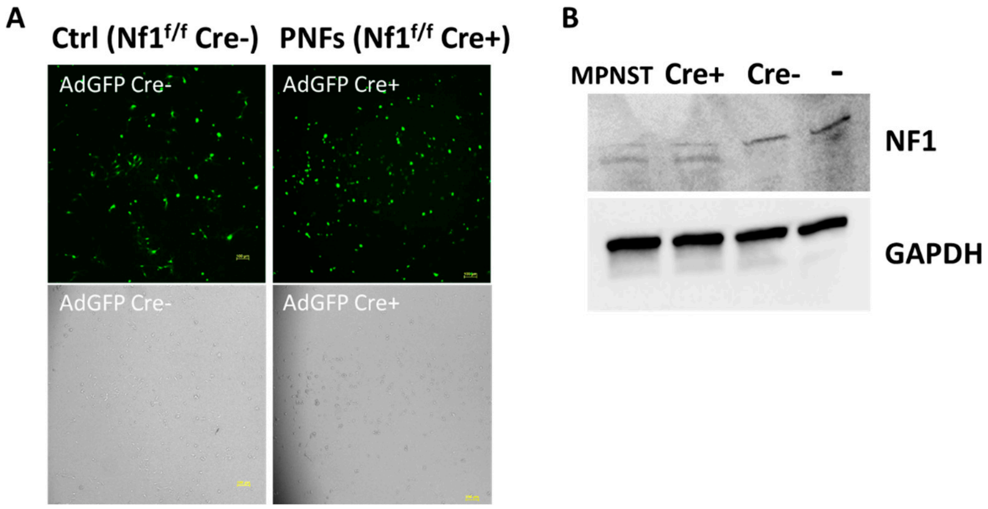

Figure S1. Knockdown efficiency of adenovirus-mediated Cre recombinase targeting Nf1 in DRG/nerve root neurosphere cells (DNSCs).

DNSCs isolated from E13.5 Nf1$^{f/f}$; PostnCre- embryos were transiently infected with a GFP-Cre adenovirus (AdGFP Cre+) to delete the Nf1 floxed alleles, generating PNF cells (Nf1$^{f/f}$ Cre+), or with a GFP adenovirus (AdGFP Cre-) as a control (Nf1$^{f/f}$ Cre-). **(A)** Fluorescence microscopy was employed to observe the efficiency of virus transduction in DNSCs. Scale bar = 100 microns. **(B)** Western blotting confirmed the Cre-mediated recombination of Nf1. '–' indicates protein lysate from DNSCs without viral infection, and MPNST was included as an Nf1-KO control.

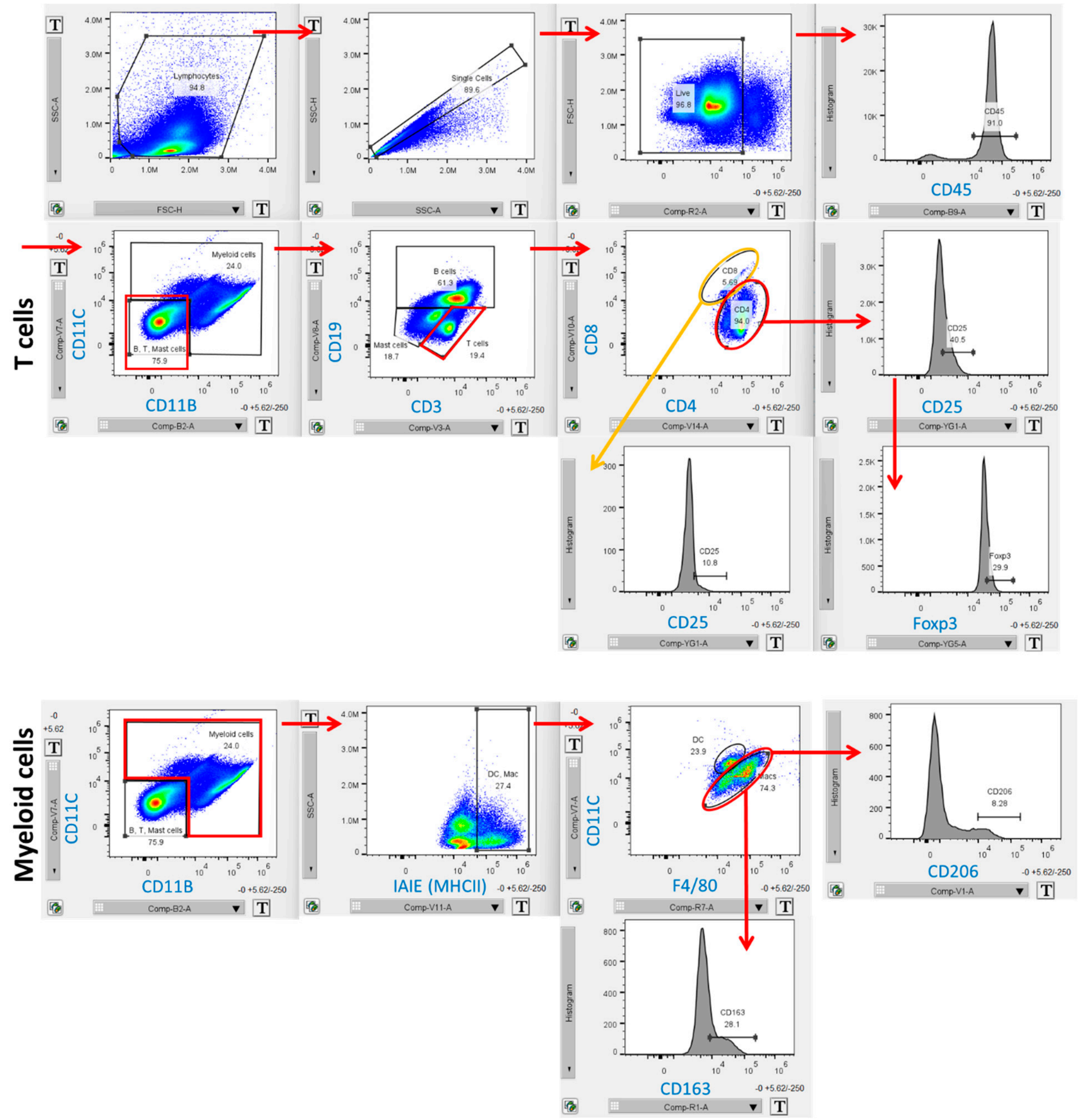

Figure S2. Gating strategy used for flow cytometry analysis of myeloid and T cell populations.
Mac= Tumor associated macrophages

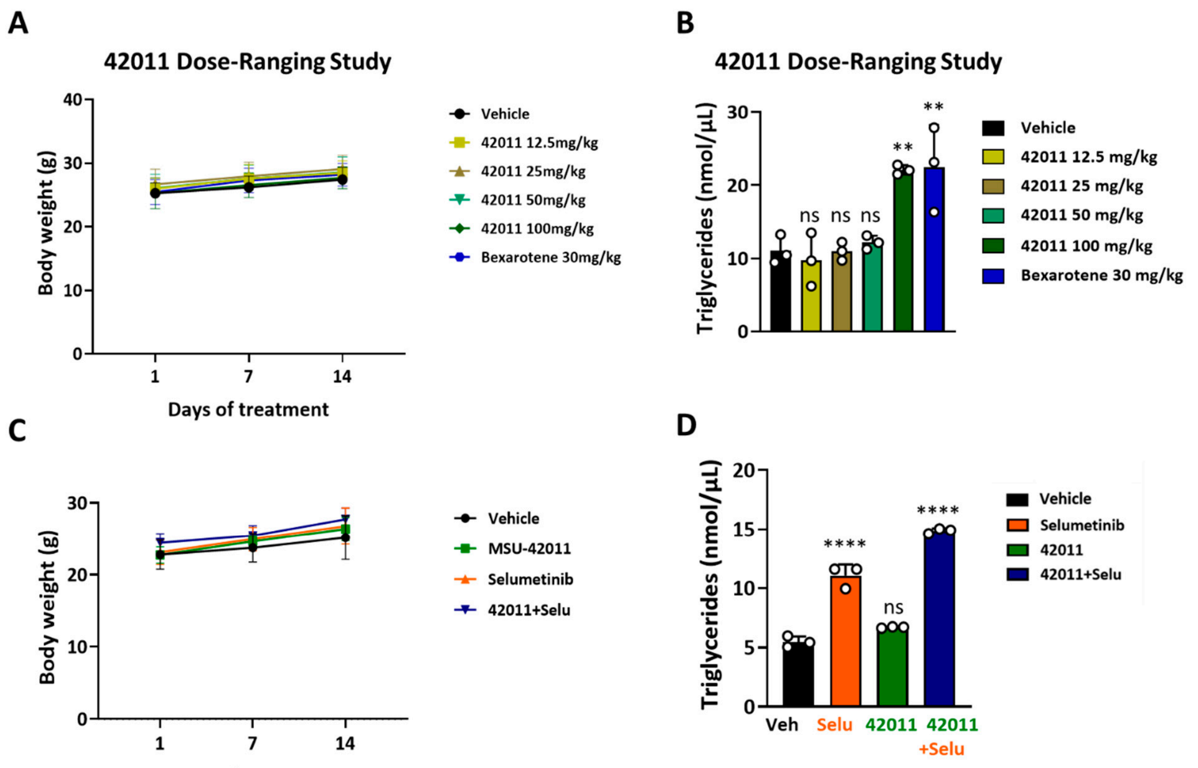

Figure S3. Triglyceride levels increased in the bexarotene and selumetinib-treated groups, but not in the MSU-42011-treated group in an immunocompetent LL2 model of lung cancer. Mouse LL2 lung cancer cells were injected into the flank of male C57BL/6 mice. Once the tumors reached 3-4 mm in diameter, mice were treated i.p. once per day, 5 days per week for 14 days, with **(A-B)** vehicle, MSU-42011 (12.5-100 mg/kg) or bexarotene (30 mg/kg); or with **(C-D)** vehicle, 25 mg/kg MSU-42011 (42011), 10 mg/kg selumetinib (Selu), or the combination. **(A, C)** Body weight was measured once per week. Data represent means ± standard deviations (n=7-9). **(B, D)** Triglyceride levels were quantified using commercially available kits as described in the methods. Data represent means ± standard deviations (n=3). ** $p < 0.01$, **** $p < 0.0001$ vs. vehicle.

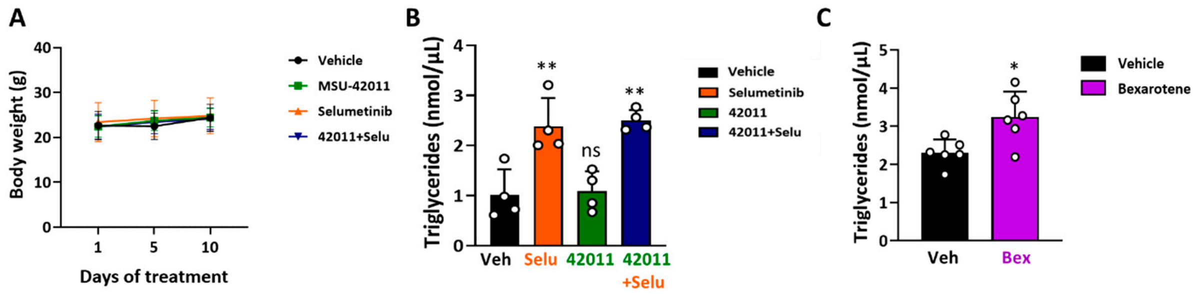

Figure S4. Triglyceride levels increased in the 10-day bexarotene and selumetinib-treated groups, but not in the MSU-42011-treated group in a mouse model of MPNST.

Mice with MPNST were treated i.p. once per day for 10 days with **(A-B)** vehicle, 25 mg/kg MSU-42011, 10 mg/kg selumetinib, the combination, or with **(C)** 25 mg/kg bexarotene. **(A)** Body weight was measured once per week. Data represent means ± standard deviations (n=12-13). **(B, C)** Triglyceride levels were quantified using commercially available kits as described in the methods. Data represent means ± standard deviations (n=4-6). * $p < 0.05$, ** $p < 0.01$ vs. vehicle.

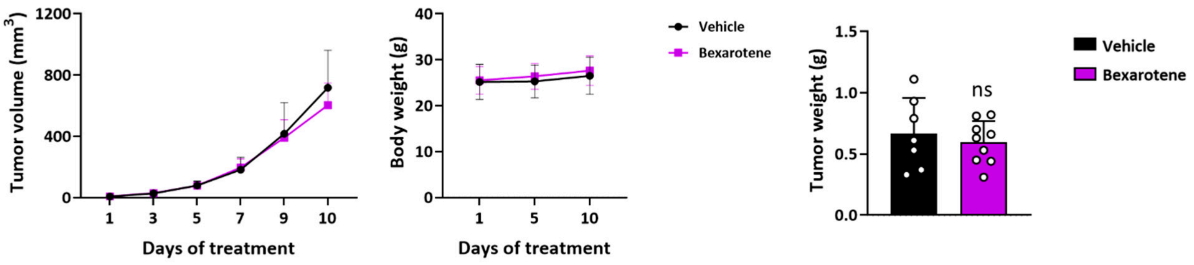

Figure S5. Bexarotene had no effect in an immunocompetent mouse model of MPNST. Mouse Nf1-related MPNST cells (mMPNST) were injected into the flank of male C57BL/6 mice. Once the tumors reached 3-4 mm in diameter, mice were treated i.p. once per day for 10 days with vehicle, 25 mg/kg bexarotene. Tumor volumes were measured by calipers every two days, and body weight was measured once per week. Data represent means ± standard deviations (n=7-9).

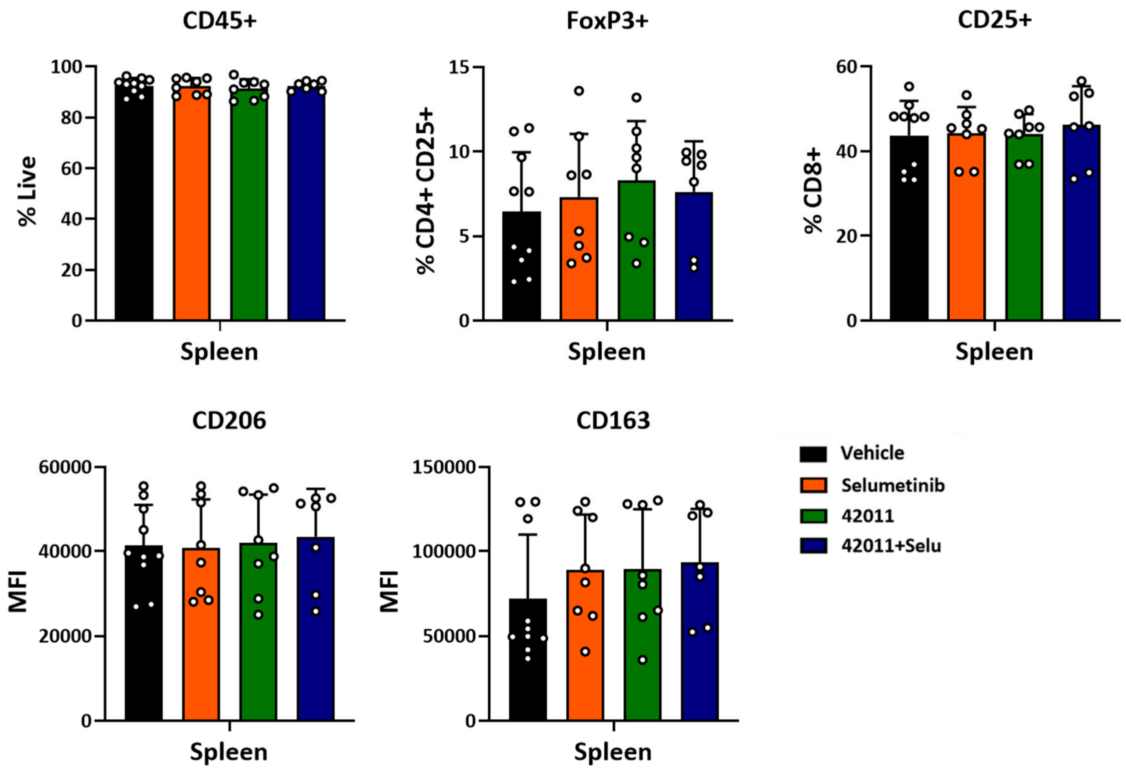

Figure S6. Immune cell populations in the spleen.

Mouse model of MPNST treated as described in figure 2. Immune cell populations within the spleen were analyzed by flow cytometry using whole spleen lysates. Data represent means ± standard deviations (n=8-10).

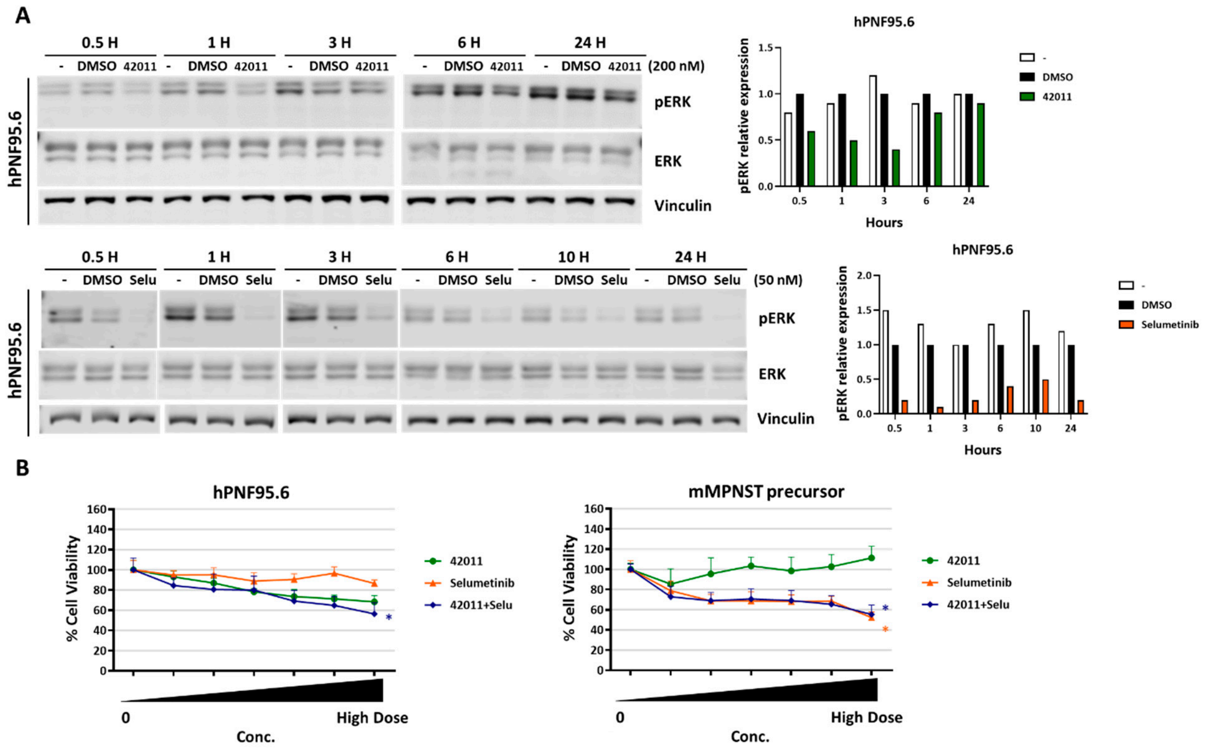

Figure S7. Effects of MSU-42011 and selumetinib on pERK levels and cell viability in NF1-deficient cells.

(A) ipNF95.6 human PNF cells (hPNF95.6) were treated with 50 nM selumetinib (Selu) and 200 nM MSU-42011 (42011) for 0.5-24 hours. The level of pERK was evaluated by western blotting and normalized to vehicle treatment at the same incubation time (n=1). **(B)** Human PNF cells (hPNF95.6) and mouse MPNST precursor cells ($Nf1^{f/f}$ $Cdkn2a^{f/f}$ Cre+) cells were treated with increasing concentrations of drugs (0-1000 nM selumetinib, 0-2000 nM MSU-42011, or the combination) for 72 hours. Cell viability was measured using an MTT assay. Data represent means ± standard deviations (n=3). * $p < 0.05$ vs. vehicle.

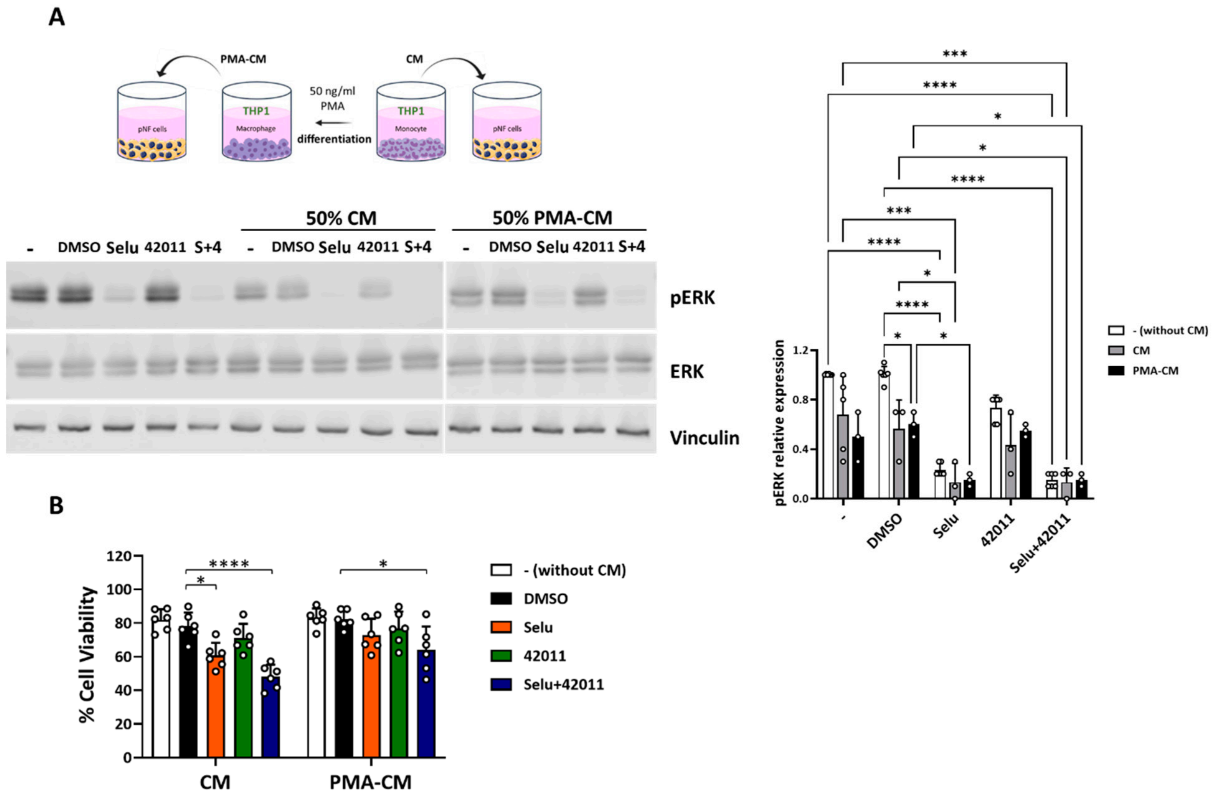

Figure S8. Conditioned media (CM) from human THP1 monocytes or macrophages did not alter pERK levels or cell viability in human PNF cells.

THP1 monocytes were treated with 50 ng/ml PMA for 3 days to differentiate into THP1 macrophages. ipNF95.6 human PNF cells were treated with 50% CM from THP1 monocytes (CM) or THP1 macrophages (PMA-CM), and treated with drugs (50 nM selumetinib, 200 nM MSU-42011, or the combination) for 3 hours to detect the level of pERK by western blotting (n=3-5) **(A)** or for 72 hours to measure cell viability by MTT assay (n=6) **(B)**. Both western blotting and MTT data were normalized to the control without CM and drug treatment (MTT data not shown). Data represent means ± standard deviations. * $p < 0.05$, **** $p < 0.0001$.

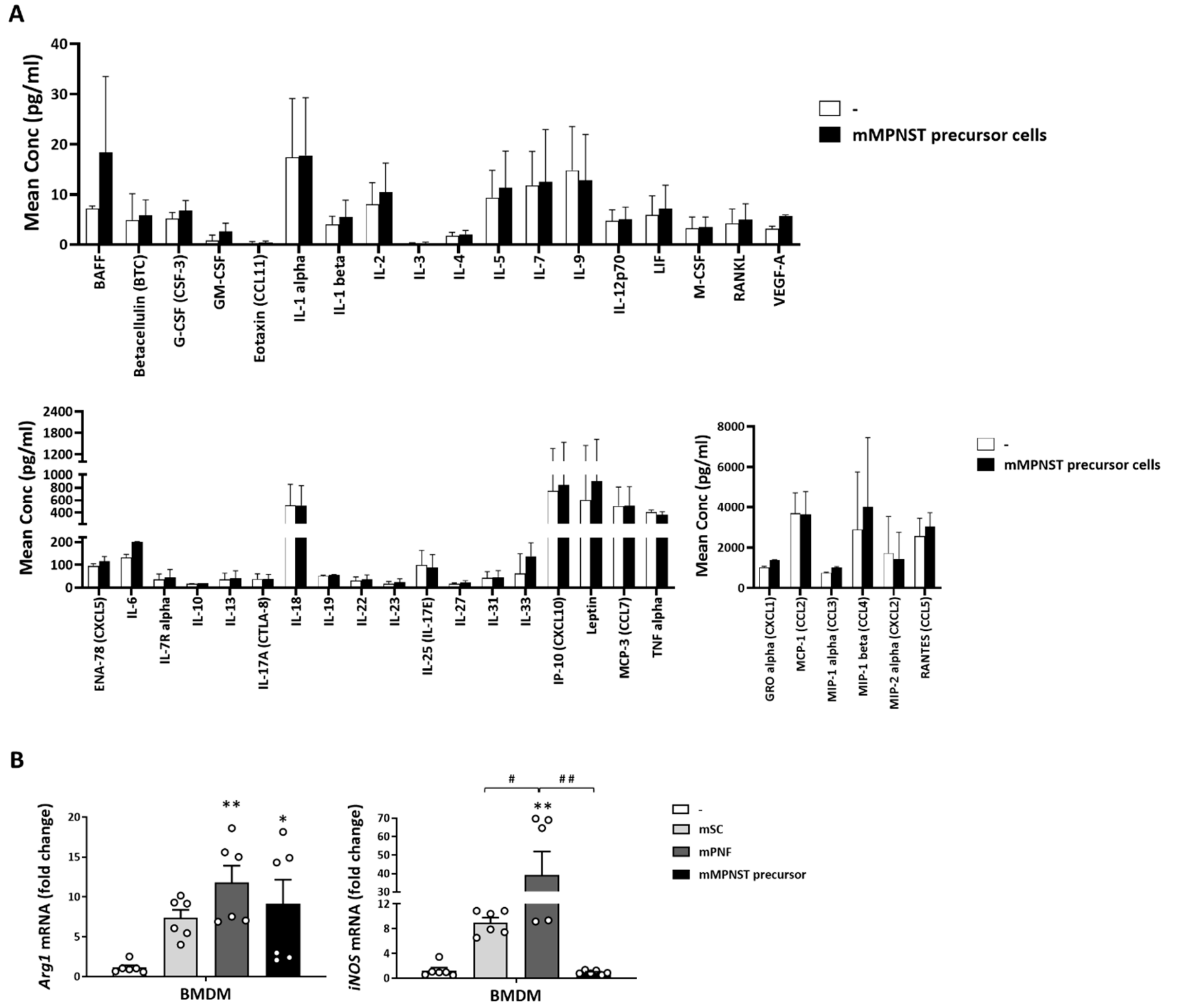

Figure S9. CM from murine MPNST precursor cells increased cytokine and chemokine secretion, while CM from murine PNF cells enhanced *Arg1* and *iNOS* mRNA expression in bone marrow-derived macrophages (BMDMs).

BMDM differentiated with 20 ng/ml M-CSF for 5 days were treated with 50% CM from mouse normal Schwann cells (Nf1$^{f/f}$ Cre-), PNF cells (Nf1$^{f/f}$ Cre+), and MPNST precursor cells (Nf1$^{f/f}$ Cdkn2a$^{f/f}$ Cre+) for 24 hours. **(A)** Supernatants from BMDM treated with CM from mouse MPNST precursor cells were analyzed by a multiplex assay. The mean concentrations were categorized as low (<20 pg/ml), medium (<1400 pg/ml), and high (<5000 pg/ml). Data were averaged across two replicates. **(B)** mRNA expressions were evaluated by qPCR and normalized to the BMDM without CM treatment. Data represent means ± standard deviations (n=5-6). * $p < 0.05$, ** $p < 0.01$ vs. BMDM without CM treatment; $^{\#}$ $p < 0.05$, $^{\#\#}$ $p < 0.01$.

Supplemental Table S1: Primer sequences used in qPCR experiments.

**Murine**	
**mGAPDH**	forward: CATCACTGCCACCCAGAAGACTG;<br>reverse: ATGCCAGTGAGCTTCCCGTTCAG
**mCCL2**	forward: CCC AAT GAG TAG GCT GGA GA;<br>reverse: AAA ATG GAT CCA CAC CTT GC
**mIL-6**	forward: TCCATCCAGTTGCCTTCTTGG;<br>reverse: CCACGATTTCCCAGAGAACATG
**mTNFα**	forward: AAGCCTGTAGCCCACGTCGTA;<br>reverse: GGCACCACTAGTTGGTTGTCTTTG
**miNOS**	forward: CTG CAG CAC TTG GAT CAG;<br>reverse: GGG AGT AGC CTG TGT GC
**mARG1**	forward: GTG AAG AAC CCA CGG TCT GT;<br>reverse: AGA AAG GAC ACA GGT TGC CC
**Human**	
**hGAPDH**	forward: GGA GCG AGA TCC CTC CAA AAT;<br>reverse: GGCTGT TGT CAT ACT TCT CATGG
**hCCL2**	forward: AGGTGACTGGGGCATTGAT;<br>reverse: GCCTCCAGCATGAAAGTCTC
**hIL-6**	forward: GGTACATCCTCGACGGCATCT;<br>reverse: GTGCCTCTTTGCTGCTTTCAC
**hTNFα**	forward: CCT GCT GCA CTT TGG AGT GA;<br>reverse: GAG GGT TTG CTA CAA CAT GGG

Supplemental Table S2: Comparison of MSU-42011 and bexarotene in mouse models of MPNST and LL2 lung cancer.

	Vehicle	MSU-42011	Bexarotene
LL2 lung cancer (14 days treatment)			
**Drug levels (mg/kg)**	None	25	30
**Efficacy-Tumor burden**			
Average tumor size (mm^3)	2536 ± 691.2	1011 ± 101.9 ****	2235 ± 269.4
**Safety-Triglyceride levels**			
Triglycerides (nmol/µL)	11.1 ± 1.1	11.0 ± 0.7	22.4 ± 3.3 **
MPNST (10 days treatment)			
**Drug levels (mg/kg)**	None	25	25
**Efficacy-Tumor burden**			
Average tumor size (mm^3)	1260 ± 155.0	377 ± 68.7 ****	
	718 ± 92.0		603 ± 48.3
Average tumor weight (g)	1.3 ± 0.2	0.5 ± 0.1 ****	
	0.7 ± 0.1		0.6 ± 0.1
**Safety-Triglyceride levels**			
Triglycerides (nmol/µL)	1.0 ± 0.3	1.1 ± 0.2	
	2.3 ± 0.1		3.2 ± 0.3 *

Mouse models were treated as described in figure 1 and 2. Values represent mean ± SE. * $p < 0.05$, ** $p < 0.01$, **** $p < 0.0001$ vs. vehicle.
Note: The comparisons between vehicle vs. MSU-42011 and vehicle vs. bexarotene in the MPNST model were performed using two independent cohorts.
