# Supplementary material for: The RXR Agonist MSU-42011 Reduces Tumor Burden in a Murine Preclinical NF1-Deficient Model
Source: Cancers (Basel). 2025 Jun 9;17(12):1920. doi: 10.3390/cancers17121920 (PMC12190937; doi:10.3390/cancers17121920)
Supplement: Supplementary file 1 [file cancers-17-01920-s001.zip › File S1.pdf]

The RXR Agonist MSU-42011 Reduces Tumor Burden in a Murine Preclinical NF1-Deficient Model

Pei-Yu Hung, Jessica A. Moerland, Ana S. Leal, Bilal Aleiwi, Edmund Ellsworth, D Wade Clapp, Verena Staedtke, Renyuan Bai, Karen T. Liby

Whole Western blot:

Figure S3A.

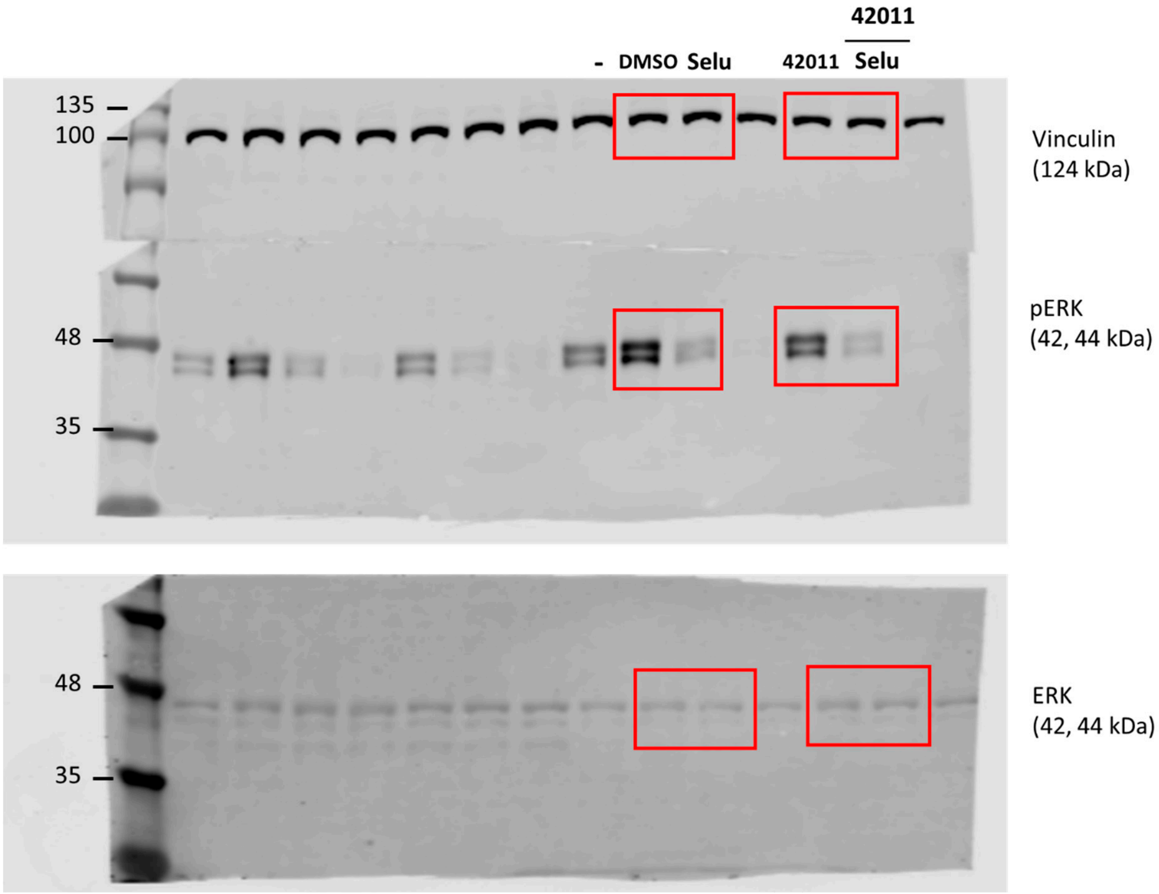

Figure S3B.

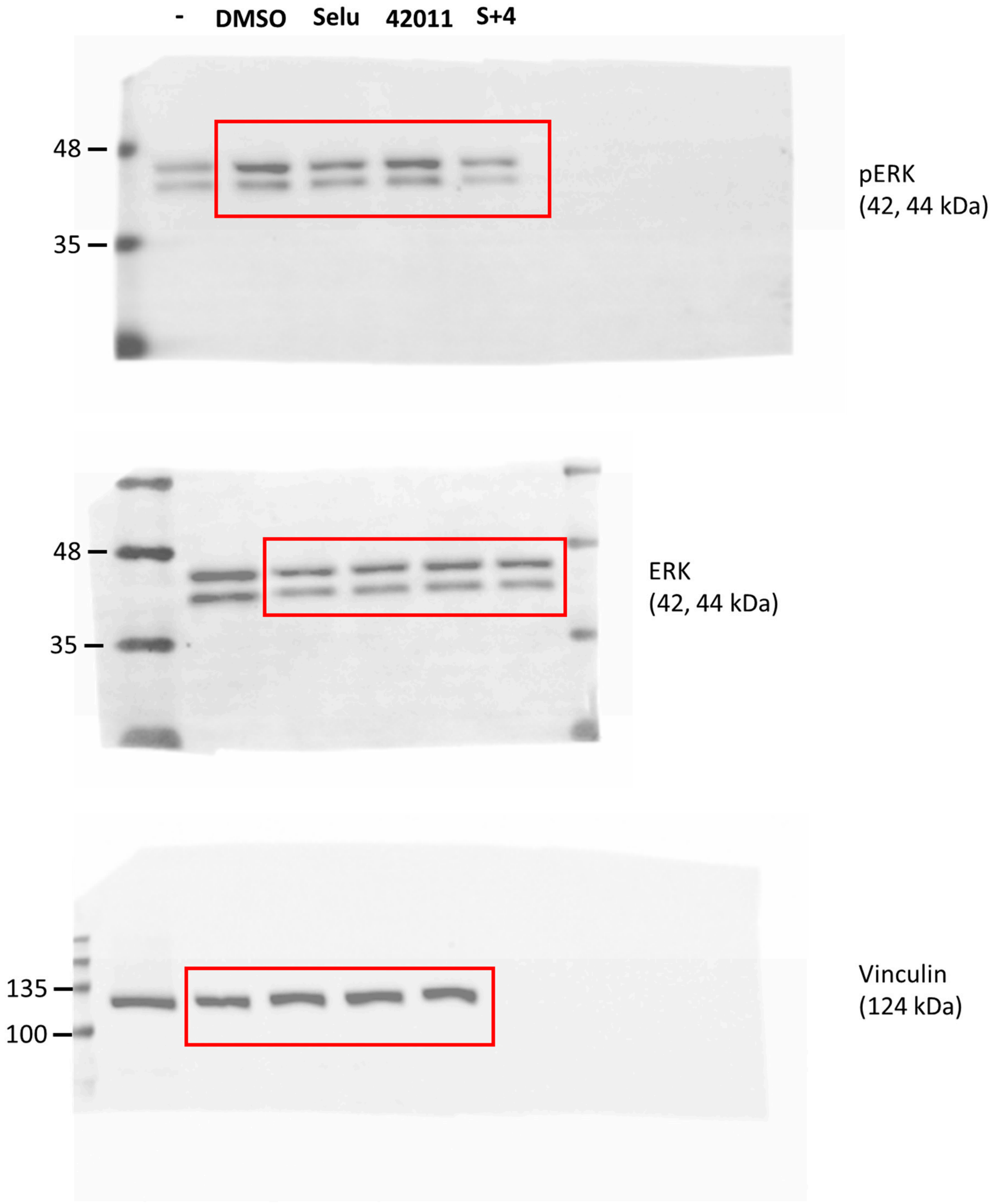

-
DMSO
Selu
42011
S+4
48
35
pERK
(42, 44 kDa)
48
35
ERK
(42, 44 kDa)
135
100
Vinculin
(124 kDa)

Figure S3C.

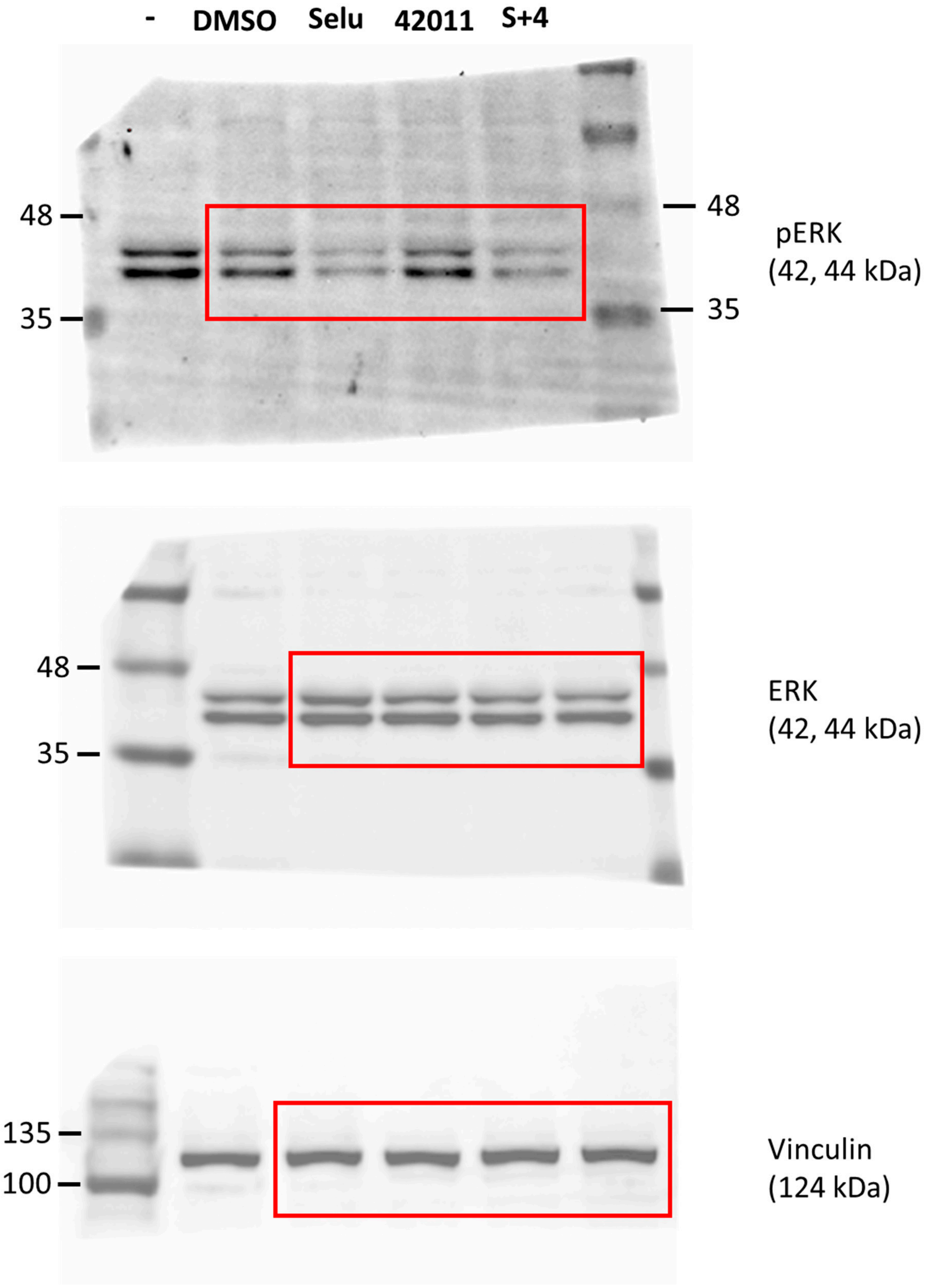
-
DMSO
Selu
42011
S+4
48
35
48
35
pERK
(42, 44 kDa)
48
35
ERK
(42, 44 kDa)
135
100
Vinculin
(124 kDa)

Figure S1B.

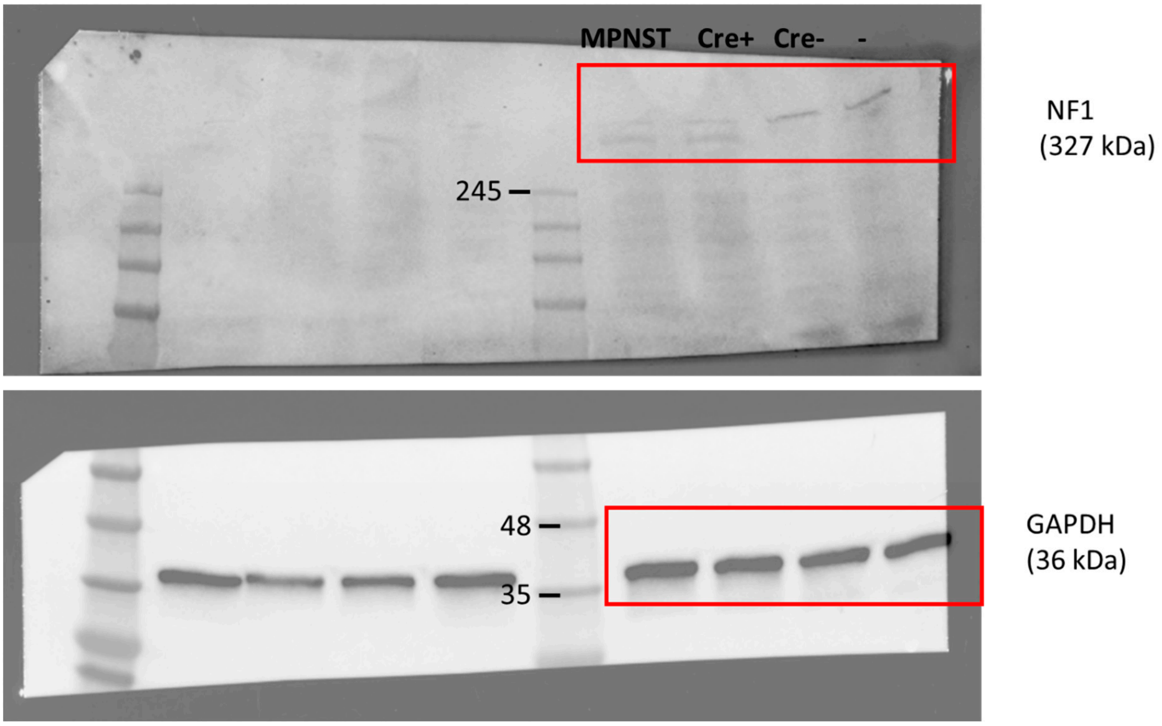

Figure S7A.

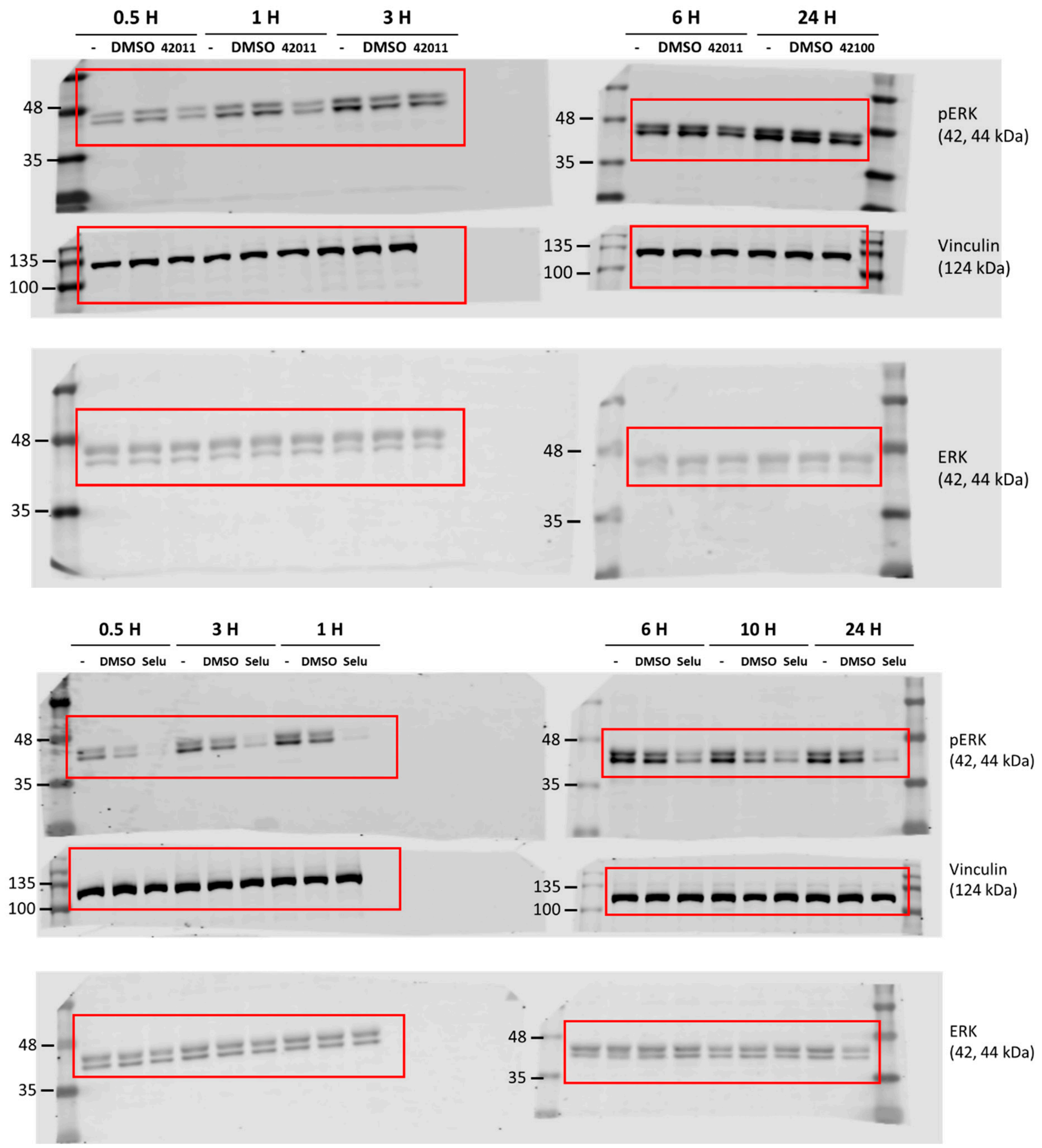
0.5 H
1 H
3 H
- DMSO 42011 - DMSO 42011 - DMSO 42011
6 H
24 H
- DMSO 42011 - DMSO 42100
48
35
135
100
pERK
(42, 44 kDa)
Vinculin
(124 kDa)
ERK
(42, 44 kDa)
0.5 H
3 H
1 H
- DMSO Selu - DMSO Selu - DMSO Selu
6 H
10 H
24 H
- DMSO Selu - DMSO Selu - DMSO Selu
pERK
(42, 44 kDa)
Vinculin
(124 kDa)
ERK
(42, 44 kDa)

18 **Figure S8A.**

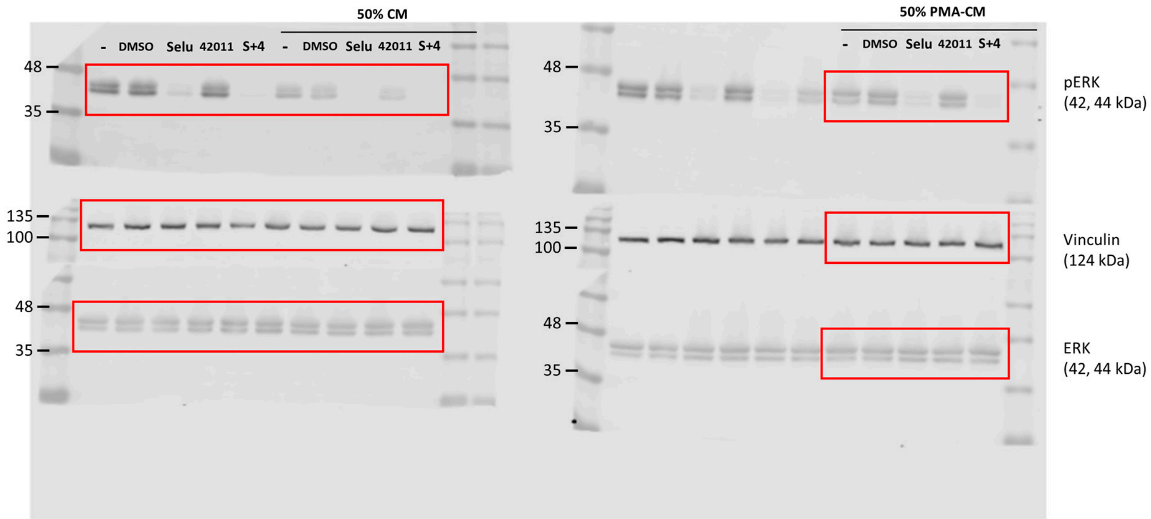

19
